# Antibody-gamma/delta T cell receptors targeting GPC2 regress neuroblastoma with low antigen density

**DOI:** 10.1016/j.xcrm.2025.102378

**Published:** 2025-09-29

**Authors:** Alex Quan, Mingyu Huo, Dan Li, Laura E. Hutchins, Constanza Rodriguez, Jangsuk Oh, Hsi-En Tsao, Madeline Spetz, Elijah Edmondson, Dana Ashworth, Rui Zheng, Jing Zhou, Jinyun Chen, Jingbao Liu, Guangyan Xiong, Hongbing Zhang, Cheng Liu, Rosa Nguyen, Nan Li, Mitchell Ho

**Affiliations:** 1 Laboratory of Molecular Biology, Center for Cancer Research, National Cancer Institute, National Institutes of Health, Bethesda, MD 20892-4264, USA; 2 Pediatric Oncology Branch, Center for Cancer Research, National Cancer Institute, National Institutes of Health, Bethesda, MD 20892-4264, USA; 3 Molecular Histopathology Laboratory, Frederick National Laboratory for Cancer Research, Frederick, MD 21702, USA; 4 Spatomics LLC, 246 Goose Ln, Ste 202A, Guilford, CT 06437, USA; 5 Eureka Therapeutics Inc., 5858 Horton Street, Suite 370, Emeryville, CA 94608, USA

**Keywords:** neuroblastoma, glypican-2, GPC2, monoclonal antibody, antibody humanization, gamma/delta TCR, antibody-TCR, AbTCR, chimeric antigen receptor, CAR, cell therapy, CD30

## Abstract

Chimeric antigen receptor (CAR) T cells have shown promise in hematological cancers but face challenges in solid tumors, partly due to heterogeneous antigen density. Glypican-2 (GPC2) is an oncofetal antigen highly expressed in neuroblastoma and under evaluation in phase 1 clinical trials. Here, we engineer T cells with antibody-T cell receptors (AbTCRs) targeting GPC2. We generate autologous AbTCR T cells using CT3 or humanized CT3 (hCT3) antigen-binding fragments (Fab) linked to γ/δ T cell receptors (TCRs), along with a CD30 co-stimulatory domain. Both CT3 and hCT3 AbTCR T cells show superior antitumor efficacy compared to CT3 CAR T cells, with hCT3 AbTCR T cells inducing significant regression in neuroblastoma with low GPC2 antigen density. Enhanced efficacy is associated with stronger TCR signaling, expansion of stem cell-like memory T cells, and improved CD8^+^ T cell infiltration. These results highlight the potential of hCT3 AbTCR T cells for neuroblastoma and indicate broad application of AbTCR T cells in solid tumors.

## Highlights

• T cells are engineered with AbTCRs by fusing antibody Fab fragments to γ/δ TCRs
• hCT3 AbTCR T cells targeting GPC2 regress neuroblastoma with low antigen density
• AbTCR efficacy is linked to improved CD8^+^ T cell infiltration and TCR signaling
• hCT3 AbTCR T cells show promising potential for neuroblastoma therapy

## Graphical abstract

**Figure:**
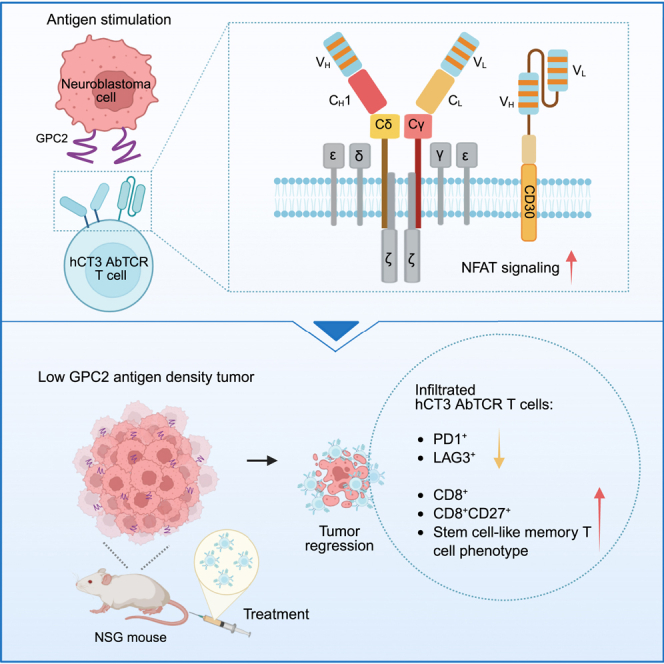

## Teaser

Quan et al. demonstrate that T cells engineered with antibody-T cell receptors (AbTCRs) targeting GPC2 outperform CAR T cells in neuroblastoma, showing improved efficacy due to stronger TCR signaling, greater memory T cell expansion, and increased CD8^+^ T cell infiltration, offering promising potential for neuroblastoma therapy.

## Introduction

Neuroblastoma is the second most frequently occurring extracranial solid tumor in children, accounting for 8%–10% of all pediatric cancers and 15% of cancer-related deaths in children.^1^ A major challenge in treating many cancers, including neuroblastoma, is the lack of effective therapeutic targets. Glypicans (e.g., GPC3) have been studied as potential targets in cancer including liver cancer.^2^^,^^3^^,^^4^ We and others identified glypican-2 (GPC2) as a target for neuroblastoma treatment.^5^^,^^6^ As an oncofetal antigen, GPC2 is expressed in neuroblastoma but is mostly absent in healthy tissue, making it an excellent target for cancer therapy.^5^ Furthermore, we found that GPC2 inhibition inactivated Wnt/β-catenin signaling and suppressed the expression of the downstream transcription factor N-Myc, an oncogenic driver of neuroblastoma tumorigenesis.^5^

Chimeric antigen receptor (CAR) T cell therapy has shown great potential in treating hematological cancers.^7^^,^^8^ However, CAR T cell therapy for solid tumors continues to face major challenges, such as low antigen density, which can result in immune escape and treatment failure.^9^^,^^10^

To overcome these limitations, various strategies have been explored to enhance the precision, potency, and persistence of CAR T cells. Recent studies have shown that c-Jun overexpression or optimization of CAR structure through iterative engineering of the hinge, transmembrane, and co-stimulatory domains can improve CAR T cell activity against tumors with low antigen density.^9^^,^^11^^,^^12^ In addition, an emerging platform known as the antibody-T cell receptors (AbTCRs) offers an alternative strategy to improve T cell functionality. The AbTCR approach combines antibody-based antigen recognition with endogenous-like T cell receptor (TCR) signaling and has demonstrated robust cytotoxicity.^13^ Furthermore, an early phase 1 clinical study reported that AbTCR T cells provided durable benefits in patients with CD19-positive relapsed/refractory B cell lymphoma.^13^^,^^14^ By preserving receptor-mediated signaling machinery of the native TCR-CD3 complex, the AbTCR T cell platform holds promise for addressing key limitations of CAR T cell therapy.

In this study, we generated T cells with engineered AbTCRs that include a synthetic antibody γδ TCR and an antibody co-stimulatory domain directing T cells to recognize and eliminate GPC2-expressing cancer cells. We previously isolated a mouse-derived monoclonal antibody (mAb) termed CT3, which binds specifically to exon 3 of *GPC2*, highly expressed in most neuroblastoma tissues with no expression in normal tissues except testis at both RNA and protein levels.^15^ The transgenic AbTCR contains a fragment antigen-binding (Fab) component, either CT3 or humanized CT3 (hCT3). The Fab is linked to TCR γ and TCR δ chains, integral parts of the naturally occurring γδ TCR. Additionally, engineered T cells incorporate a single-chain fragment variable (scFv)-CD30 fusion as a co-stimulatory domain, activated upon GPC2 recognition, enhancing TCR signaling in T cells. We propose that AbTCR T cells may exhibit greater activity compared to CAR T cells against GPC2^low^ tumors due to their lower antigen threshold for receptor activation. This feature enables AbTCR T cells to potentially overcome challenges associated with low antigen levels, often encountered in patients with heterogeneous expression and those who have undergone unsuccessful CAR T cell therapy.

## Results

### hCT3 CAR T cells suppress neuroblastoma growth in mice

**Figure 1:**
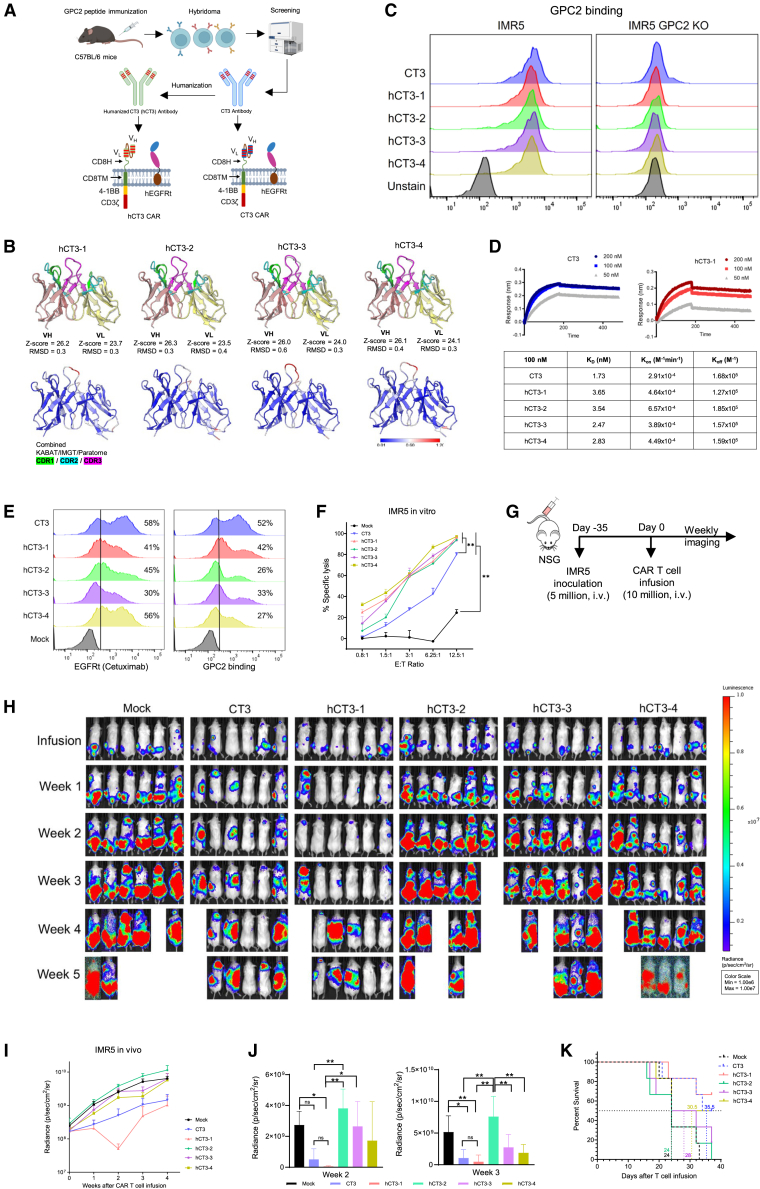
Antitumor efficacy of hCT3 CAR T cells against IMR5 neuroblastomas (A) CT3 and hCT3 antibodies were developed through hybridoma technology and CDR grafting. The schematics of CT3 and hCT3 CAR T cells are illustrated using BioRender.com. (B) Structural models of humanized CT3 antibodies were aligned with the original CT3 antibody model (shown in blue). (C) The binding affinity of antibodies was assessed via flow cytometry. (D) Octet analysis evaluated GPC2 binding by CT3 and hCT3 antibodies. K_D_, Kon, and Koff values at 100 nM concentration are tabulated. (E) CAR expression and GPC2 binding of CAR T cells. Mock group was stained only with the secondary antibody. (F) Cytolytic activity of CAR T cells against IMR5 cells was measured at various effector-to-target (E:T) ratios. *n* = 3. *p* values were calculated at the E:T ratio of 12.5:1. (G) *In vivo* study schematic: NSG mice were inoculated with 5 million IMR5 cells and treated via tail vein injection. *n* = 6 mice. (H) Representative bioluminescence images of tumor-bearing mice were obtained using IVIS imaging. (I and J) Tumor growth curves of the IMR5 mouse model (H) until the end of the experiment (I) and group comparison at week 2 and week 3 (J). (K) Kaplan-Meier survival curve. The dashed line marks median survival. *n* = 6. Values represent mean ± SEM. ^ns^*p* > 0.05, ∗*p* < 0.05, ∗∗*p* < 0.01. See also Figures S1 and S2.

Our lab previously isolated a GPC2-specific mouse CT3 mAb through hybridoma technology and generated CT3 CARs.^15^ Here, to reduce antibody immunogenicity in humans, we humanized CT3 mAb through complementarity-determining region (CDR) grafting and structural modeling (Figure 1A). Four human single-chain variable fragment (scFv) backbones were selected to generate hCT3 variants (Figure S1). We used AlphaFold3^16^ to generate structural models of CT3 and its four humanized forms (hCT3-1 to hCT3-4) and aligned them to assess structural integrity (Figure 1B). The heavy- and light-chain variable regions (VH and VL) from the four humanized CT3 antibodies shared similar *Z* scores (23.5–26.3) with low overall root-mean-square deviation (RMSD) (0.3–0.6). Only hCT3-3 exhibited the highest local RMSD at the VH CDR3 region (0.6), suggesting structural variation at its antigen-binding site due to humanization. The hCT3 mAbs showed similar binding to the GPC2-expressing neuroblastoma cell line IMR5, with no binding to the IMR5 GPC2 knockout cell line (Figure 1C). Octet BLI analysis further validated the binding, and the similar K_D_ values indicated comparable binding between the hCT3 mAbs and the CT3 mAb (Figures 1D and S2A–S2C), suggesting that CDR grafting preserved the binding capacity of the CT3 mAb while implementing a humanized framework.

With the humanized antibodies retaining comparable binding to GPC2, we generated four CAR T cells using the scFv derived from hCT3 mAbs. The structures of both the CT3 and hCT3 CAR T cells (Figure 1A) included a CD8 hinge (CD8H), CD8 transmembrane domain, a 4-1BB costimulatory domain, and a CD3ζ T cell signaling domain. A truncated human epidermal growth factor receptor was included to allow for tracking of the CAR T cells and to act as an off switch, as previously described.^17^ We measured CAR T cells’ transduction efficiency and GPC2 binding capacity. The hCT3-1 CAR T cells exhibited the highest GPC2 binding ability (42%) among the four hCT3 CAR T cells tested, while transduction efficiencies were comparable (41%) (Figure 1E).

All hCT3 CAR T cells exhibited significantly increased cytolytic activity, with nearly 100% specific lysis against the neuroblastoma cell line IMR5 compared to CT3 CAR T cells (80%) at an effector to target (E:T) ratio of 12.5:1 (*p* < 0.01) (Figures 1F and S2D). To compare the antitumor activity of CAR T cells *in vivo*, we used the metastatic IMR5 intravenous (i.v.) xenograft mouse model. Mice received either 10 million mock T or CAR T cells when the bioluminescence of the tumor reached 2 × 10^8^ photons/s/cm^2^/sr (Figure 1G). Interestingly, hCT3-1 CAR T cells demonstrated the highest antitumor efficacy compared to CT3 and the other hCT3 CAR T cells, with 33% (2 out of 6) of mice experiencing 100% tumor regression by week 3 post infusion. In contrast, no mice in the CT3, hCT3-2, -3, and -4 CAR T cell groups achieved 100% tumor clearance by week 3 (Figures 1H–1J). At the experiment’s endpoint, 83% (5 out of 6) of mice survived in the CT3 and hCT3-1 CAR groups, while only two mice (33%) survived in the hCT3-2 CAR group, which had a median survival of 24 days. Additionally, three mice (50%) survived in the hCT3-3 and hCT3-4 CAR groups, both with a median survival of 28 days (Figure 1K). Based on these data, we concluded that hCT3-1 is the most suitable humanized CT3 variable fragment sequence and decided to use it for further preclinical development. To simplify terminology, we will henceforth refer to it as “hCT3” in future experiments.

### Enhanced activity of hCT3 AbTCR T cells in a metastatic neuroblastoma mouse model

**Figure 2:**
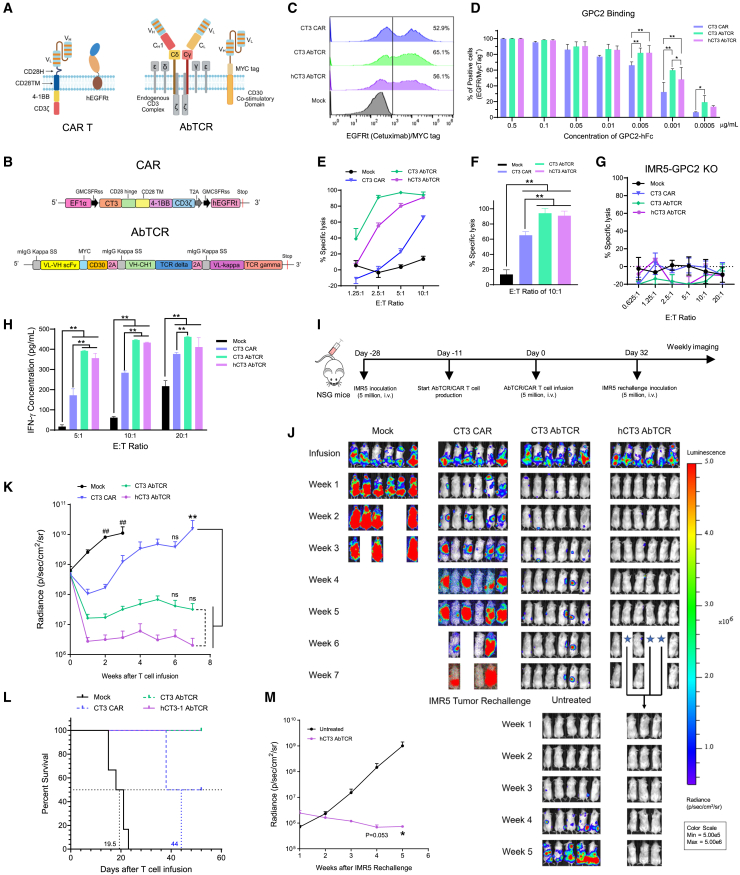
AbTCR T cells targeting GPC2 induced complete regression of metastatic IMR5 neuroblastomas in mice (A) Diagrams of GPC2-targeted CAR and AbTCR T cell constructs are illustrated using BioRender.com. (B) Schema of the CT3 CAR and AbTCR construct. (C) CAR expression was measured using flow cytometry. (D) CAR and AbTCR Jurkat T cells were incubated with GPC2-hFc to assess T cell binding via flow cytometry. *n* = 3. (E–G) Cytolytic activity of CAR and AbTCR T cells against IMR5 (E and F) and IMR5-GPC2 knockout (KO) cells (G) was assessed. *n* = 3. (H) IFN-γ levels in co-cultured supernatants with IMR5 cells. (I) *In vivo* study schematic. (J–L) Tumor bioluminescence images (J), tumor growth curves (K), and Kaplan-Meier plot of survival curve (L) of mice treated with CAR or AbTCR T cells. *n* = 6. ##*p* < 0.01 compared to CAR and AbTCR groups. (M) Tumor growth curves of IMR5 cell-rechallenged mice in untreated and hCT3 AbTCR T cell-treated groups. *n* = 3–5. *p* values were calculated at weeks 4 and 5. Values represent mean ± SEM. ^ns^*p* > 0.05, ∗*p* < 0.05, ∗∗*p* < 0.01. See also Figure S3.

To devise a GPC2-targeted cell therapy effective against low GPC2-expressing tumors, we decided to develop AbTCR T cells based on the CT3 and hCT3 mAbs. This scaffold uses γ and δ TCR chains to engage the endogenous CD3 complex for T cell signaling. The AbTCR T cell platform comprises two separate complexes: the γδTCR complex, which utilizes a Fab for antigen binding, and a CD30-based co-stimulatory domain that uses the GPC2 scFv for co-stimulatory signaling (Figures 2A and 2B). The Fab was intentionally designed to retain only the CH1 domain to maintain proper folding and stability. We constructed CT3 AbTCR and hCT3 AbTCR T cells by integrating CT3 and hCT3 Fab into the AbTCR format, respectively (Figures 2A and 2B). Meanwhile, we improved the CT3 CAR T cell by replacing CD8HTM with CD28HTM (Figures 2A, 2B, S3A, and S3B). CT3 CAR T cells with the CD28HTM demonstrated enhanced antitumor efficacy both *in vitro* and in the IMR5 i.v. mouse model (Figures S3C–S3F). This design is supported by a previous study demonstrating that the CD28 hinge and transmembrane domains enhance CAR function in a low-antigen-density leukemia cell line (NALM6).^9^ CT3 CD28HTM, the most potent CAR construct we have developed for targeting GPC2-expressing neuroblastoma, is currently being developed for a clinical trial. Therefore, we used CT3 CD28HTM CAR T cells as the comparator for evaluating the AbTCR T cell platform in the following experiments.

We found similar transduction efficiencies of CT3 CAR (52.9%), CT3 AbTCR (65.1%), and hCT3 AbTCR (56.1%) T cells by staining with anti-EGFRt cetuximab or anti-MYC tag (Figure 2C); however, their ability to bind GPC2 varied (Figure 2D). Beginning at the 0.01 μg/mL concentration of GPC2-hFc, the CT3 CAR T cell began losing their ability to bind GPC2 as only 77% of cells remained bound to GPC2. Even at lower concentrations (0.0005 to 0.005 μg/mL), both AbTCR constructs demonstrated higher binding capacity to GPC2 compared to CT3 CAR T cells (Figure 2D). The increased GPC2 binding observed in both CT3 and hCT3 AbTCR T cells may, in part, be attributed to structural differences in surface receptor composition. Unlike the CAR system, which features a single antigen-binding domain, the AbTCR platform co-expresses two GPC2-binding components: the Fab-based AbTCR and the scFv-CD30 co-receptor, potentially enhancing overall binding avidity for the antigen. This structural distinction may contribute to the stronger binding signal observed in the AbTCR groups. Both CT3 and hCT3 AbTCR T cells showed significantly increased cytolytic activity against IMR5 cells compared to CT3 CAR T cells, with AbTCR T cells achieving approximately 90% specific lysis, while CT3 CAR T cells only reached 65% specific lysis at an E:T ratio of 10:1 (Figures 2E and 2F). None of the T cells exhibited killing activity in GPC2 knockout IMR5 cells (Figure 2G). Moreover, CT3 and hCT3 AbTCR T cells induced 1.5- to 2-fold higher interferon-gamma (IFN-γ) secretion compared to CT3 CAR T cells upon stimulation with IMR5 cells at E:T ratios of 5:1 and 10:1 (*p* < 0.01) (Figure 2H). Next, we examined the antitumor effects of CT3 CAR, CT3 AbTCR, and hCT3 AbTCR T cells using a metastatic IMR5 mouse model (Figure 2I). Mice were treated when the bioluminescence signal reached approximately 1 × 10^9^ photons/s/cm^2^/sr following IMR5 infusion. This contrasts with the similar mouse model shown in Figure 1G, in which treatment was initiated at a smaller tumor burden, when the bioluminescence signal reached approximately 3 × 10^8^ photons/s/cm^2^/sr. The groups treated with CT3 AbTCR and hCT3 AbTCR T cells experienced rapid tumor regression. They demonstrated significantly decreased tumor burden at week 7 post infusion compared to mock and CT3 CAR T cell-treated groups (Figures 2J and 2K). Notably, the hCT3 AbTCR T cells induced complete regression of IMR5 neuroblastoma xenografts within 1 week, and all the mice in this group remained tumor-free until week 7. In contrast, only 16% (1 out of 6) of mice in the CT3 CAR T cell group and 66% (4 out of 6) of mice in the CT3 AbTCR T cell group experienced complete tumor regression. At week 7, 100% overall survival was observed in both the CT3 and hCT3 AbTCR T cell groups, whereas only 50% (3 out of 6) of the mice in the CT3 CAR T cell group survived, and no survivors remained in the mock group (Figure 2L). To investigate the persistence of hCT3 AbTCR T cells, we rechallenged tumor-free mice with IMR5 tumor cells at week 6 post infusion. As shown in Figures 2J and 2M, the hCT3 AbTCR T cell treatment group maintained complete tumor regression for up to 5 weeks (*p* < 0.05), indicating the long-term persistence of hCT3 AbTCR T cells in the mice.

### Enhanced antitumor efficacy of hCT3 AbTCR T cells in a high-GPC2 neuroblastoma subcutaneous mouse model

**Figure 3:**
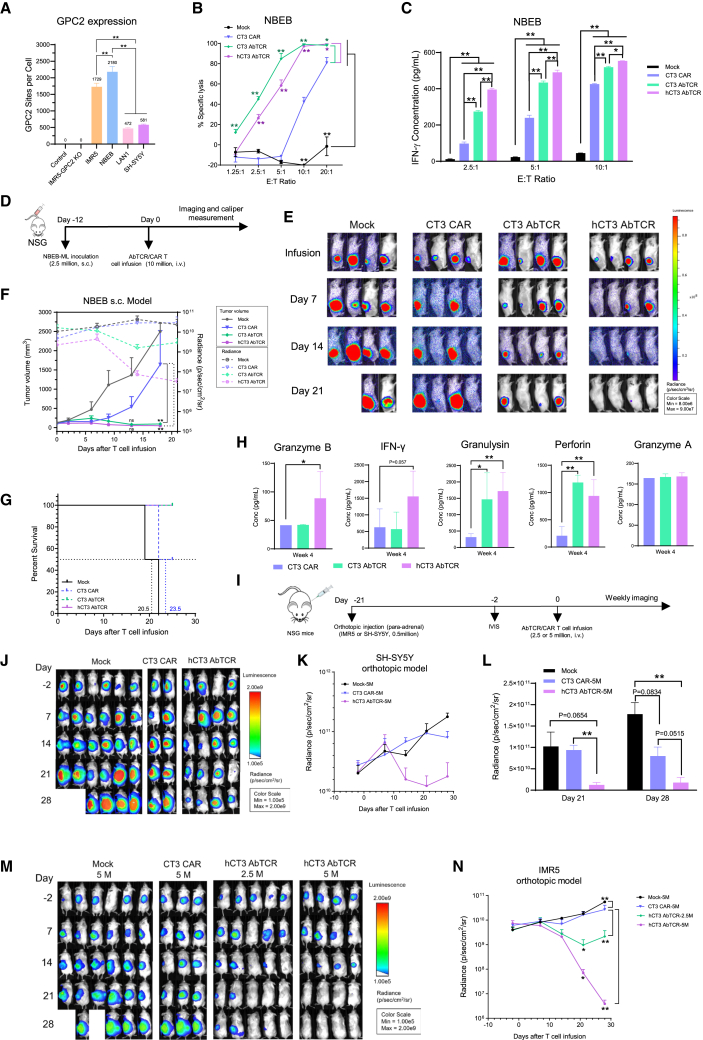
Enhanced antitumor efficacy of hCT3 AbTCR T cells in subcutaneous and orthotopic mouse models (A) GPC2 expression levels in cells. Control means the IMR5 with secondary antibody staining. *n* = 3. (B) Cytolytic activity of CAR and AbTCR T cells against NBEB tumor cells. *n* = 3. (C) IFN-γ levels in co-cultured supernatants with NBEB tumor cells. *n* = 3. (D) Experimental schema of the NBEB xenograft mouse model. (E–G) Tumor bioluminescence images (E), tumor volume (F), and Kaplan-Meier survival curve (G) of mice treated with CAR or AbTCR T cells. Dual-axis chart was used in (F): solid lines indicate tumor volumes measured by caliper (left *y* axis), and dashed lines indicate radiance measured by IVIS (right *y* axis). *n* = 4. (H) Plasma levels of cytokines in NBEB mice at week 4 of CAR or AbTCR T cell infusion. *n* = 6. (I) *In vivo* study schematic. (J–L) Tumor bioluminescence images (J), tumor growth curve (K), and statistical analysis (L) of orthotopic SH-SY5Y mouse cohorts. *n* = 2–6. (M and N) Tumor bioluminescence images (M) and tumor growth curve (N) of orthotopic IMR5 mouse cohorts. *n* = 3–6. Values represent mean ± SEM. ^ns^*p* > 0.05, ∗*p* < 0.05, ∗∗*p* < 0.01.

We further compared the antitumor efficacy of CAR and AbTCR T cells in the NBEB cell line, which showed relatively high GPC2 expression with 2,180 molecules per cell (Figure 3A). CT3 and hCT3 AbTCR T cells killed nearly 100% of NBEB cells at E:T ratios of 10:1 and 20:1, respectively, whereas CT3 CAR T cells achieved only 40%–80% cytotoxicity (Figure 3B). In addition, NBEB cells exhibited reduced cytotoxic sensitivity compared to IMR5 cells (Figure 2E vs. Figure 3B), despite expressing higher levels of GPC2 (2,180 vs. 1,729 sites/cell). Both CT3 and hCT3 AbTCR T cells demonstrated enhanced IFN-γ secretion levels compared to CT3 CAR T cells at E:T ratios of 2.5:1, 5:1, and 10:1 (*p* < 0.01). Notably, the secretion level of IFN-γ in hCT3 AbTCR T cells was significantly higher than in CT3 AbTCR T cells (Figure 3C).

We then conducted a heterotopic xenograft neuroblastoma mouse model using subcutaneously injected NBEB tumor cells (Figure 3D). The hCT3 AbTCR T cell-treated group exhibited a remarkable 94.01% reduction in tumor size by day 14 post infusion. Tumor volumes in both the CT3 and hCT3 AbTCR groups regressed significantly by day 18 post infusion compared to CT3 CAR and mock groups. At the experiment’s endpoint, hCT3 AbTCR T cells demonstrated a 96.31% tumor reduction with 100% overall survival. In contrast, the CT3 AbTCR T cells resulted in a 79.15% tumor reduction, while only two mice in the CT3 CAR group showed a tumor regression of 69.36%, accompanied by a 50% overall survival rate. This indicates the significantly enhanced antitumor activity of hCT3 AbTCR T cells (Figures 3E–3G). Furthermore, we evaluated cytokine levels in the NBEB mouse model. Consistent with cell-killing potency, both CT3 and hCT3 AbTCR T cell-treated groups showed higher secretion of granulysin and perforin (*p* < 0.05 or *p* < 0.01) and similar levels of IFN-γ and granzyme A at week 4 post infusion. The hCT3 AbTCR T cell group also exhibited enhanced levels of granzyme B (*p* < 0.05) (Figure 3H).

### hCT3 AbTCR T cells showed enhanced antitumor efficacy in both high- and low-antigen orthotopic mouse models

To further validate the therapeutic efficacy of hCT3 AbTCR T cells in more physiologically relevant models, we used two orthotopic mouse models with para-adrenal injections of 0.5 million IMR5 cells and low-antigen-expressing SH-SY5Y cells (581 sites/cell), followed by the administration of CAR T cells or hCT3 AbTCR T cells (2.5 million or 5 million) at a higher tumor signal (around 1 × 10^10^ photons/s/cm^2^/sr) (Figure 3I). In the established low-antigen orthotopic mouse model, the hCT3 AbTCR T cell group regressed the tumor better than CT3 CAR T cell, with 3 out of 4 mice showing partial responses and a mean regression of 80.45% at day 28 post infusion (Figures 3J–3L). We further tested two treatment doses in the IMR5 orthotopic mouse model, including 5 million and a lower dose of 2.5 million. Both hCT3 AbTCR T cell groups receiving these two doses showed significant tumor regression compared to the CT3 CAR group starting on day 21 post infusion. The group treated with 5 million hCT3 AbTCR T cells demonstrated a 99.96% tumor reduction at the end of the experiment (Figures 3M and 3N). These findings demonstrate that GPC2-targeted AbTCR T cells are more effective than CAR T cells in treating neuroblastoma, with hCT3 AbTCR T cells showing the highest activity.

### hCT3 AbTCR T cells eliminate low-antigen-density tumors and promote T cell infiltration

**Figure 4:**
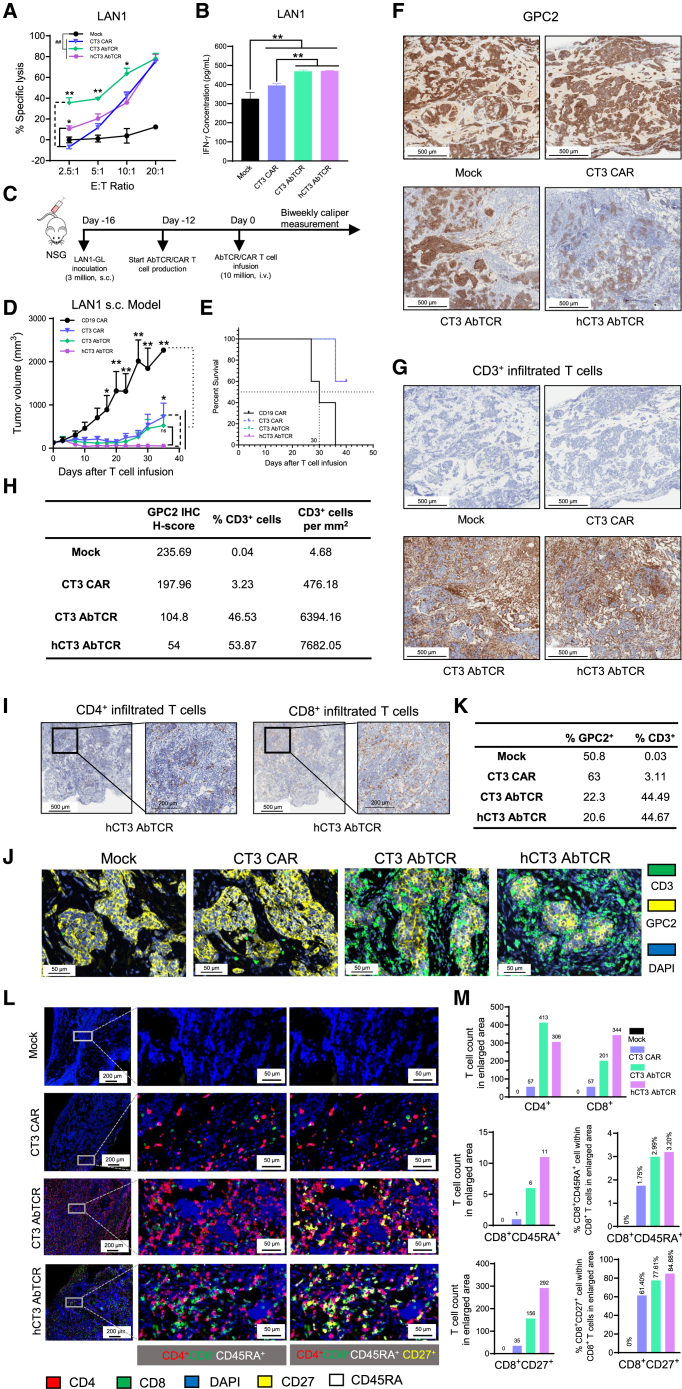
hCT3 AbTCR T cell caused significant regression of LAN1 neuroblastoma xenografts with low antigen density in mice (A) Cytolytic activity of CAR and AbTCR T cells against LAN1 tumor cells. *n* = 3. ∗*p* < 0.05, ∗∗*p* < 0.01 compared to CT3 CAR T cells; ^##^*p* < 0.01 compared to mock at the E:T ratios of 10:1 and 20:1. (B) IFN-γ levels in co-cultured supernatants with LAN1 cells at an E:T ratio of 10:1. *n* = 3. (C–E) Experimental schema (C), tumor volume (D), and Kaplan-Meier survival curve (E) of the LAN1 xenograft mouse model. *n* = 5. (F–H) Representative immunohistochemistry staining images for GPC2 (F) and CD3 (G) in tumor sections and corresponding quantification (H) from the LAN1 mouse model. Scale bars: 500 μm. (I) Immunohistochemical detection of CD4- and CD8-positive cells in tumor sections from LAN1 mouse model. Scale bars: 200 or 500 μm. (J and K) Representative immunofluorescence staining images (J) and corresponding quantification (K) in tumor sections from the LAN1 mouse model. Scale bars: 50 μm. (L and M) Multiplexed immunofluorescence in tumor sections from the LAN1 mouse model (L) and corresponding quantification results (M). Scale bars: 50 or 200 μm. Values represent mean ± SEM. ^ns^*p* > 0.05, ∗*p* < 0.05, ∗∗*p* < 0.01; ^##^*p* < 0.01. See also Figure S4.

To further examine the antitumor activity of CAR and AbTCR T cells in low-antigen-density tumors, we utilized the LAN1 cell line, which has about 472 molecules per cell (Figure 3A), to perform *in vitro* and *in vivo* experiments. Cell-killing assays showed that CT3 AbTCR T cells performed better than CT3 CAR T cells at E:T ratios of 5:1 and 10:1 (*p* < 0.05 or *p* < 0.01). At the lower E:T ratio of 2.5:1, both CT3 and hCT3 AbTCR T cells demonstrated higher specific lysis than CT3 CAR T cells against LAN1 cells (Figure 4A). We also detected IFN-γ secretion levels in the co-cultured supernatant. The results showed that both CT3 and hCT3 AbTCR T cells promoted IFN-γ secretion levels compared to the CT3 CAR and mock groups (*p* < 0.01) (Figure 4B). Subsequently, we evaluated the antitumor activity of CAR and AbTCR T cells using the LAN1 xenograft mouse model (Figure 4C). Mice treated with hCT3 AbTCR T cells showed a significantly reduced tumor burden and a comparable survival curve compared to the CT3 CAR group by day 35 (*p* < 0.05) (Figures 4D and 4E), suggesting that hCT3 AbTCR T cells are ideal for treating low-GPC2-antigen-density tumors.

To investigate the infiltration of T cells in tumor tissues, we employed immunohistochemistry (IHC) staining to detect the expression of antigen (GPC2) (Figure 4F) and CD3^+^ (Figure 4G) T cells in tissue sections from the LAN1 mouse model. As the tissue was collected at an early treatment time point to allow histological analysis, residual tumor presence was expected as shown in Figure 4F. The groups treated with AbTCR T cells demonstrated significantly lower GPC2 expression, with the hCT3 AbTCR T cell group showing the lowest tumor cell staining intensity (H-score 54.0) compared to the mock (H-score 235.69) and CT3 CAR groups (H-score 197.96). Furthermore, CT3 and hCT3 AbTCR T cell groups demonstrated 46.53% and 53.87% of infiltrated CD3^+^ T cells, respectively, which is higher than CT3 CAR (3.23%) and mock groups (0.04%) (Figures 4F–4H). Meanwhile, the CD3^+^ cell number (per mm^2^) in the CT3 (6394.16) and hCT3 (7682.05) AbTCR T cell groups was much higher than in the mock (4.68) and CT3 CAR (476.18) groups (Figure 4H). The remarkable expansion of tumor-infiltrating lymphocytes, combined with fewer tumor cells per unit area, aligned with the observed tumor reduction in the hCT3 AbTCR T cell group. Importantly, there was a notable increase in CD8^+^ T cells compared to CD4^+^ T cells among the infiltrated T cells in the hCT3 AbTCR T cell-treated group (Figure 4I).

Consistent with the IHC results, immunofluorescence staining indicated that CT3 and hCT3 AbTCR T cell groups had lower GPC2 expression, with values of 22.3% and 20.6%, respectively, compared to 50.8% in the mock group and 63% in the CT3 CAR group. Additionally, the percentage of CD3^+^ cells was higher in the CT3 (44.49%) and hCT3 (44.67%) AbTCR T cell groups compared to the mock (0.03%) and CT3 CAR T cell (3.11%) groups (Figures 4J, 4K, and S4). Consistent with the IHC findings, immunofluorescence staining revealed that CD8^+^ T cell infiltration was markedly elevated in the hCT3 AbTCR T cell group (344 cells) compared to the CT3 AbTCR (201 cells) and CT3 CAR T cell groups (57 cells). Notably, the hCT3 AbTCR T cell group exhibited the highest percentage of activated CD8^+^CD27^+^ T cells at 84.44%, followed by CT3 AbTCR (77.61%) and CT3 CAR (61.4%) T cells. Moreover, the proportion of CD8^+^CD45RA^+^ cells was slightly higher in the hCT3 AbTCR T cell group (3.2%) relative to CT3 CAR (1.75%) and CT3 AbTCR (2.99%) T cells (Figures 4L and 4M).These findings suggest that the hCT3 AbTCR T cell group elicited a stronger cytotoxic T cell response, characterized by higher CD8^+^ T cell infiltration and a greater proportion of activated CD8^+^CD27^+^ T cells within the tumor microenvironment.

### Enhanced stemness and reduced exhaustion in hCT3 AbTCR T cells promote antitumor activity and persistence in mice

**Figure 5:**
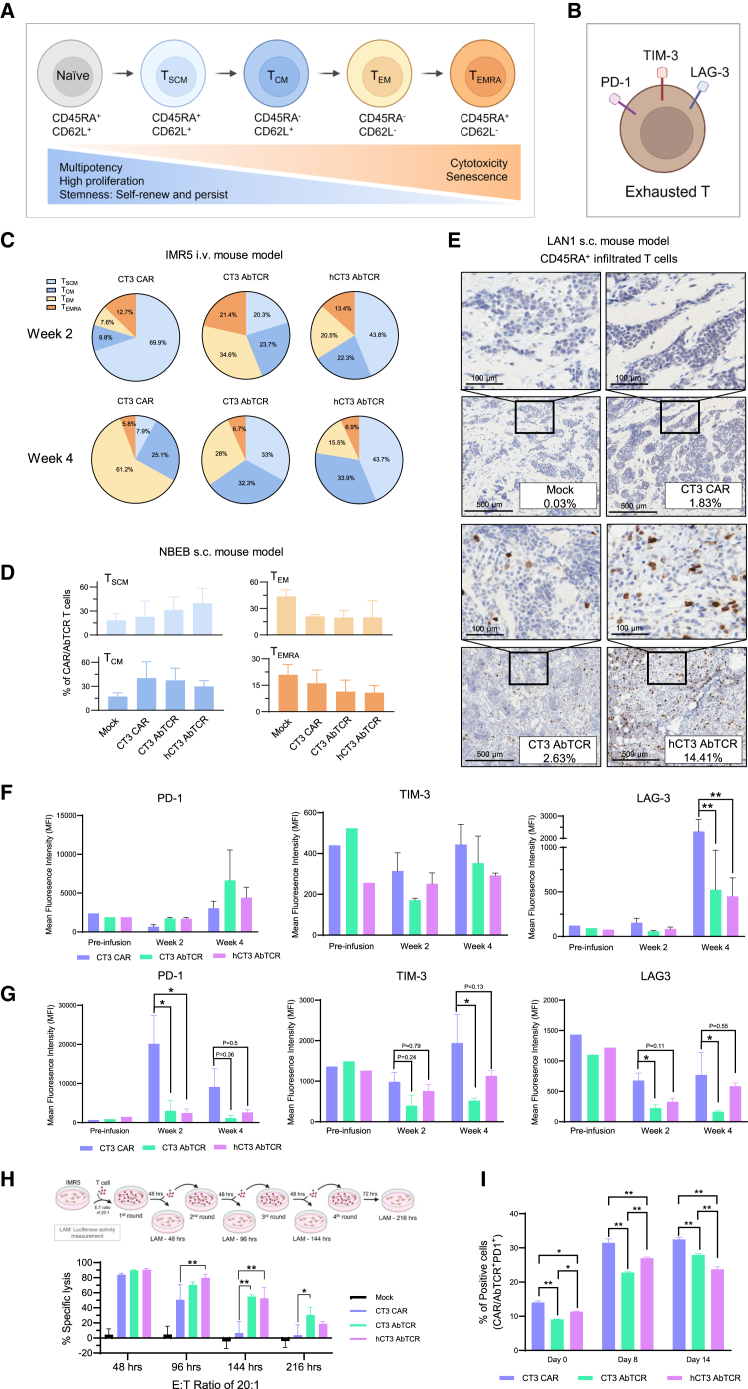
Elevated stem cell-like memory phenotype and reduced exhaustion markers in AbTCR T cells in mice (A and B) The schematic models for the differentiation of circulating memory T cell subsets (A) and exhausted T cells (B) were illustrated using BioRender.com. (C and D) Distribution of blood memory T cell subsets in the IMR5 i.v. mouse model (C), and at week 2 post infusion in the NBEB subcutaneous (s.c.) mouse model (D). (E) Representative immunohistochemistry staining images in tumor sections from the LAN1 s.c. mouse model and quantification of the percentage of CD45RA^+^ cells. Scale bars: 100 or 500 μm. (F and G) Flow cytometry analysis of exhaustion markers levels in CAR and AbTCR T cells from the IMR5 i.v. mouse model (F) and NBEB s.c. mouse model (G). *n* = 3 mice/group. (H) Four rounds of cytolytic activity of CAR and AbTCR T cells against IMR5 tumor cells at an E:T ratio of 20:1. *n* = 3. (I) Flow cytometry analysis of CAR/AbTCR^+^PD-1^+^ T cell percentage after co-culturing with NBEB cells. *n* = 3. Values represent mean ± SEM. ^ns^*p* > 0.05, ∗*p* < 0.05, ∗∗*p* < 0.01.

To investigate the factors contributing to the enhanced antitumor activity and prolonged persistence of hCT3 AbTCR T cells, we analyzed circulating CAR T and AbTCR T cells in IMR5 and NBEB mouse models. Different surface marker expressions define T cell states from naive to terminally differentiated effector memory T cells (T_EMRA_) (Figure 5A)^18^ and associate with functional exhaustion in T cells (Figure 5B). At week 2 post infusion in the IMR5 mouse model, we observed a higher percentage of effector memory T (T_EM_) and central memory T (T_CM_) cells and a lower percentage of stem cell-like memory T (T_SCM_) cells in CT3 AbTCR T cells (T_EM_: 34.6%, T_CM_: 23.7%, and T_SCM_: 20.3%) and hCT3 AbTCR T cells (T_EM_: 20.5%, T_CM_: 22.3%, and T_SCM_: 43.8%) compared to CT3 CAR T cells (T_EM_: 7.6%, T_CM_: 9.8%, and T_SCM_: 69.9%). This suggests that AbTCR T cells have a higher fraction of cytolytic effectors than CAR T cells (Figure 5C). By week 4, the percentage of T_SCM_ cells was significantly increased in CT3 AbTCR T cells (33%) and hCT3 AbTCR T cells (43.7%) compared to CT3 CAR T cells (7.9%), indicating that AbTCRs, particularly hCT3 AbTCR T cells, can self-renew and differentiate into effector T cells more efficiently *in vivo* over an extended period (Figure 5C). At 4 weeks, CT3 CAR T cell exhibited a higher fraction of T_EM_, suggesting that these CAR effectors terminally differentiated in the context of tumor persistence.

In the NBEB mouse model, treatment with CT3 and hCT3 AbTCR T cells also resulted in a significantly higher percentage of T_SCM_ cells and a lower percentage of T_EM_ cells than CT3 CAR T cells at 2 weeks post adoptive T cell injection. Specifically, the total T_SCM_ cell population was 31.3% in CT3 AbTCR T cells and 39.5% in hCT3 AbTCR T cells, compared to 22.6% in CT3 CAR T cells. Meanwhile, the T_EM_ cell population was 19.8% in CT3 AbTCR T cells and 19.9% in hCT3 AbTCR T cells, compared to 21.1% in CT3 CAR T cells (Figure 5D). We also evaluated the expression of CD45RA in the low-antigen-density LAN1 tumors. Figure 5E showed that the percentage of CD45RA^+^ cells in hCT3 AbTCR T cells is 14.41%, which is much higher than in the CT3 CAR (1.83%) and CT3 AbTCR (2.63%) T cell groups.

Additionally, we examined the expression of exhaustion markers, including programmed cell death protein 1 (PD-1), T-cell immunoglobulin and mucin domain 3 (TIM-3), and lymphocyte activation gene 3 (LAG-3) (Figure 5B), in circulating CAR and AbTCR T cells. Both CT3 and hCT3 AbTCR T cells exhibited decreased LAG-3 expression (*p* < 0.01) and comparable levels of PD-1 and TIM-3 to CAR T cells at week 4 post infusion in the IMR5 mouse model (Figure 5F). Similarly, in the NBEB mouse model, CT3 AbTCR T cells showed lower expression levels of TIM-3 at week 4 and LAG-3 at weeks 2 and 4 post infusion compared to CT3 CAR T cells (*p* < 0.05) (Figure 5G). Both CT3 and hCT3 AbTCR T cells exhibited significantly decreased expression levels of PD-1 at week 2 post infusion (Figure 5G). Furthermore, we conducted a four-round killing assay at an E:T ratio of 20:1 to compare the efficacy of CAR and AbTCR T cells after prolonged antigen exposure (Figure 5H). The efficacy of both CAR and AbTCR T cells decreased as the duration extended; however, both AbTCR T cells exhibited better killing activity compared to CT3 CAR T cells during the third round (144 h), and even in the fourth round (216 h), CT3 AbTCR T cells maintained significantly better efficacy than CT3 CAR T cells (Figure 5H). To assess T cell exhaustion following chronic antigen exposure, we co-cultured NBEB tumor cells with T cells at a 1:1 ratio, transferring the T cells to a newly passaged NBEB-plated dish with the same 1:1 ratio every 48 h. On days 8 and 14, both AbTCR T cells maintained lower PD1 expression than CT3 CAR T cells (Figure 5I). This indicates that both AbTCR constructs exhibited improved effector function and experienced less exhaustion than CT3 CAR during chronic antigen exposure *in vivo*.

Collectively, the immunophenotypic analysis of persistent transgenic T cells in the IMR5, NBEB, and LAN1 neuroblastoma models demonstrates that hCT3 AbTCR T cells regressed tumor through the enrichment of stemness and the reduction of inhibitory immune checkpoints.

### Upregulation of the T cell signaling pathway by hCT3 AbTCR T cells

**Figure 6:**
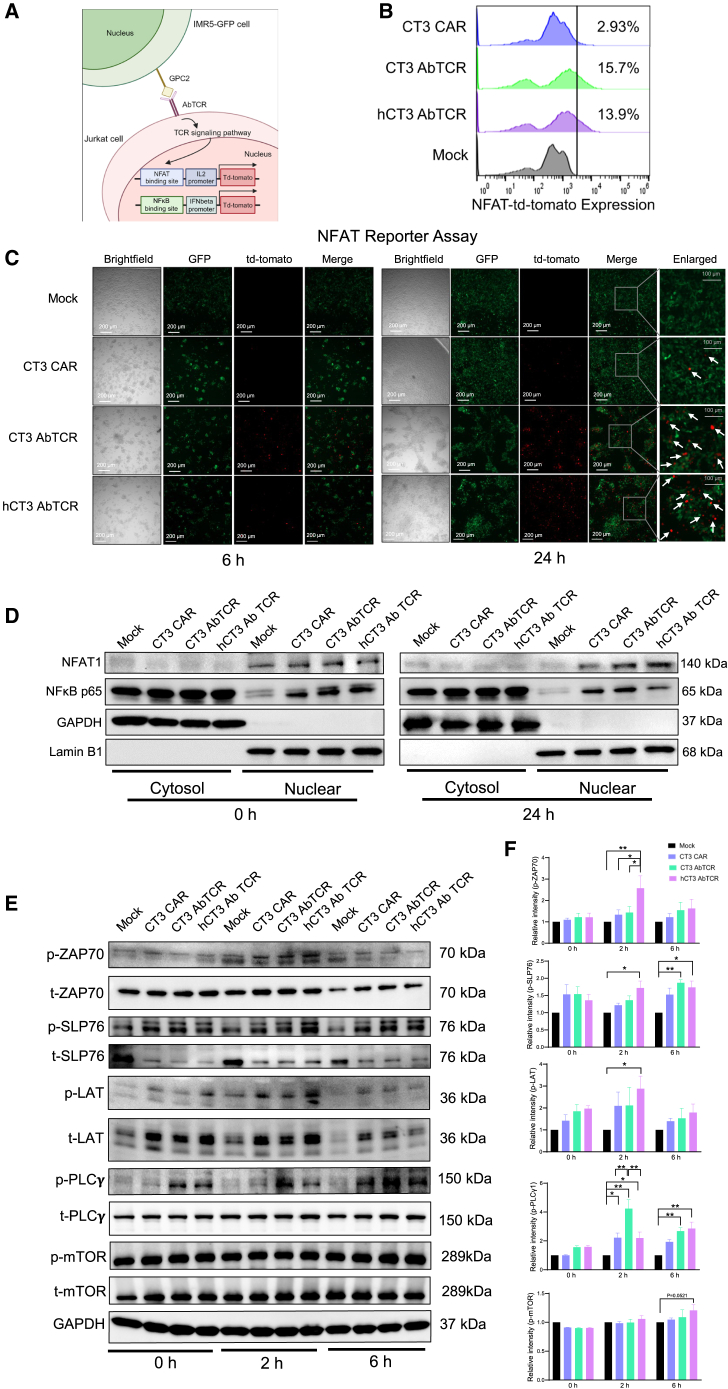
hCT3 AbTCR T cells enhanced activation of TCR signaling via the NFAT pathway in T cells (A) Schematic of the NFAT reporter assay. (B) NFAT-td-tomato expression levels were measured at 24 h. (C) Confocal microscopy images of NFAT Jurkat reporter cells interacting with IMR5 tumor cells. Scale bars: 200 μm. White arrow: Td-tomato. (D) Nuclear translocation levels of NFAT1 and NF-κB in CAR and AbTCR T cells after co-culturing with tumor cells. *n* = 3. (E and F) Representative western blot images (E) and summary data (F) showing TCR signaling in CAR and AbTCR T cells after co-culturing with NBEB tumor cells. *n* = 3. Values represent mean ± SEM. ∗*p* < 0.05, ∗∗*p* < 0.01. See also Figure S5.

AbTCR T cells utilize γδ TCR chains to engage with the endogenous CD3 complex via receptor-mediated T cell activation, differing from CAR T cells that covalently link a CD3ζ domain as an activation domain in their CAR construct. We further investigated the downstream signaling pathways in activated CAR and AbTCR T cells to determine whether the superior antitumor efficacy of AbTCR T cells is due to enhanced signaling pathways. We generated a Jurkat-td-tomato NFAT line and a Jurkat-td-tomato nuclear factor κB (NF-κB) reporter line to measure NFAT and NF-κB activity through co-culturing with IMR5 cells expressing green fluorescent protein (Figure 6A). Using flow cytometry, we found that the expression of NFAT-td-tomato in CT3 and hCT3 AbTCR T cells was 15.7% and 13.9%, respectively, higher than in CT3 CAR T cells (2.93%) after 24 h of co-culture (Figure 6B). Similarly, the fluorescence microscopy data indicated higher NFAT-td-tomato levels in both CT3 and hCT3 AbTCR T cells (Figure 6C). We also found that the nuclear translocation of NFAT1 was elevated in both AbTCR T cells, especially hCT3 AbTCR T cells, compared to CT3 CAR T cell and mock groups (Figure 6D). However, the expression of NF-κB-td-tomato was comparable in CT3 CAR and CT3/hCT3 AbTCR T cells (0.56%–2.06%) (Figures S5A and S5B). Similarly, the nuclear translocation of NF-κB-p65 was comparable between the CT3 CAR and CT3 AbTCR T cell groups, with a slightly decreased level in the hCT3 AbTCR T cell group (Figure 6D).

Further investigation revealed that the TCR signaling pathway was significantly activated in hCT3 AbTCR T cells after co-culturing with NBEB tumor cells for 2 h compared to mock, CT3 CAR, and CT3 AbTCR T cells, with increased expression of phosphorylated ZAP70, SLP76, and LAT (Figures 6E and 6F). PLCγ1 was also significantly activated in all CAR and AbTCR T cells compared to mock at 2 h. In contrast, only CT3/hCT3 AbTCR groups showed continued activation of PLCγ1 and SLP76 at 6 h (*p* < 0.05 or *p* < 0.01), indicating that both AbTCR T cells can sustain TCR signaling activation in the short term compared to CT3 CAR T cells. We also examined mammalian target of rapamycin (mTOR) activity, which supports proliferation and survival; however, we did not find a significant difference in mTOR phosphorylation across all groups after activation for 2 and 6 h (Figures 6E and 6F). Overall, this suggests that hCT3 AbTCR T cells can more robustly engage NFAT to activate the endogenous TCR signaling pathway compared to CAR T cells in the context of tumor cells.

## Discussion

In this study, we constructed AbTCR T cells targeting GPC2 by fusing the Fab domain of CT3 or hCT3 mAb with the γ and δ chains of the TCR to activate T cells via receptor-mediated CD3 activation. We also co-expressed the scFv of CT3 or hCT3 fused to the CD30 co-stimulatory domain to enhance TCR signaling upon antigen recognition. This AbTCR scaffold recognizes antigen in a human leukocyte antigen-independent manner, like CARs, but requires a much lower antigen threshold than CARs to activate endogenous TCR signaling for cytotoxicity. Accordingly, AbTCR T cells induced rapid and enhanced regression of IMR5 and NBEB neuroblastoma xenografts (1,729–2,180 GPC2 molecules per cell) in mice within 1 or 2 weeks. Notably, hCT3 AbTCR T cells caused significant tumor regression of low-antigen-density neuroblastoma, including the subcutaneous mouse model using LAN1 (472 GPC2 molecules per cell) and the orthotopic mouse model using SH-SY5Y (581 GPC2 molecules per cell). The superior efficacy of hCT3 AbTCR T cells was related to a higher percentage of T_SCM_ and T_CM_, more robust NFAT signaling, decreased expression of inhibitory immune checkpoint molecules, and increased infiltration of activated CD8^+^ T cells into the tumor microenvironment. This work suggests that hCT3 AbTCR T cells could be beneficial for patients with naturally low levels of GPC2 in their tumors or those with GPC2-CAR-resistant neuroblastoma.

Harris et al. reported that engineered TCR-T cells with fused antibodies recognizing peptide-major histocompatibility complex (MHC) complexes showed greater antigen sensitivity than CARs,^19^^,^ indicating potential benefits by utilizing endogenous TCR signaling. However, TCR-T cell therapy is limited to MHC-presented targets, despite promising clinical results.^20^^,^^21^ The two-chained AbTCR T cell platform integrates antibody-based antigen recognition with the engagement of the native CD3 complex composed of three dimers (εγ, εδ, and ζζ) that interact with the intracellular domains of the γ/δ chains.^22^ This interaction resembles endogenous TCR signaling, differing from conventional CAR T cells that depend on synthetic activation domains. In the present study, AbTCR T cells showed enhanced cytotoxicity, likely due to activation of native T cell pathways involving CD3-mediated phosphorylation and NFAT1-driven cytokine gene expression.

Using mouse models, we found that AbTCR T cells showed superior antitumor activity compared to CAR T cells. In the IMR5 model, AbTCR T cells had a higher percentage of T_EM_ cells by week 2, causing rapid tumor regression. While CAR T cell-treated mice showed tumor relapse, AbTCR T cells maintained tumor control and achieved 100% remission upon rechallenge, indicating that AbTCR T cells retained durable T cell memory. This functional persistence correlated with higher T_SCM_ and T_CM_ levels at week 4 (Figure 5C). In contrast, CAR T cells displayed more T_EM_ but fewer T_SCM_ and T_CM_ cells, suggesting functional exhaustion and reduced capacity for sustained antitumor response. Furthermore, the hCT3 AbTCR group showed a predominance of CD8^+^ T cells, particularly activated CD8^+^CD27^+^ T cells (Figures 4L–4M), suggesting that CD8^+^ hCT3 AbTCR T cells may play a critical role in tumor regression. Future studies employing peripheral depletion of CD8^+^ T cells in immunocompetent mouse models could provide direct validation of their role in mediating antitumor activity.

Heitzeneder et al. reported a strategy to enhance GPC2-CAR efficacy against low-antigen-density neuroblastoma by engineering the transmembrane and co-stimulatory domains and overexpressing c-Jun.^12^ The AbTCR T cell platform operates through a distinct mechanism engaging the native TCR-CD3 complex to enable more sensitive and physiologically regulated activation. These approaches are not mutually exclusive and may be combined to further improve therapeutic outcomes.

Additionally, although the subcutaneous LAN-1 model used in this study serves as a stringent model for evaluating T cell infiltration, future studies will incorporate a patient-derived xenograft mouse model that more accurately reflects the heterogeneity and antigen expression profiles of human neuroblastoma.

In summary, we constructed the hCT3 AbTCR based on the humanized CT3 antibody targeting GPC2-positive solid tumors. This study highlights the superior antitumor efficacy of the AbTCR T cell platform and its potential clinical utility of GPC2-targeted hCT3 AbTCR T cells for treating neuroblastoma. This approach may help address key challenges associated with CAR T therapy in solid tumors, which can lead to insufficient antigen recognition and suboptimal T cell activation. While the current study demonstrates robust efficacy in immunotherapy-naive mouse models, further research is needed to evaluate the therapeutic potential of hCT3 AbTCR T cells in tumors that relapse or develop resistance to conventional CAR T therapy. The functional and therapeutic analysis of the hCT3 AbTCR T cell may provide insights into understanding and optimizing receptor-mediated signaling activation for T cell-based cancer therapies. We believe the AbTCR T cell scaffold may also serve as a promising platform for developing therapies targeting other tumor-associated antigens in solid tumors.

### Limitations of the study

In future work, it will be useful to directly compare AbTCR T cells with CAR T cells incorporating CD30 co-stimulation to determine whether the enhanced efficacy arises from CD30 or synergistic integration with the AbTCR architecture.

## Resource availability

### Lead contact

Further information and requests for resources should be directed to and will be fulfilled by the lead contact, Mitchell Ho (homi@mail.nih.gov).

### Materials availability

All unique/stable reagents generated in this study are available from the lead contact with a completed materials transfer agreement.

### Data and code availability

• All data relevant to the study are included in the article or uploaded as supplemental information.
• No custom computer code was generated in this study.
• Any additional information required to reanalyze the data reported in this work is available from the lead contact upon request.

## Acknowledgments

This research was supported in part by the Intramural Research Program of the 10.13039/100000002National Institutes of Health (10.13039/100000002NIH) (Z01 BC010891 and ZIA BC010891 to M. Ho). The contributions of the 10.13039/100000002NIH authors were made as part of their official duties as 10.13039/100000002NIH federal employees, are in compliance with agency policy requirements, and are considered Works of the United States Government. However, the findings and conclusions presented in this paper are those of the authors and do not necessarily reflect the views of the NIH or the U.S. Department of Health and Human Services. We thank the NCI CCR Animal Resource Program/NCI Biological branch for providing the NSG mice used in the present study, 10.13039/100000054NCI CCR/Leidos Animal Facility for assisting in animal support, the 10.13039/100000050NHLBI Biophysics Core for assistance in antibody kinetics/affinity analysis, 10.13039/100000054NCI CCR Confocal Microscopy Core for assistance in confocal microscopic analysis, NCI CCR Flow Cytometry Core Facility for assistance in cellular staining, and 10.13039/100012728Frederick National Laboratory for Cancer Research Molecular Histopathology Laboratory for tissue staining service. 10.13039/100012728Frederick National Laboratory for Cancer Research was funded in whole or in part with federal funds from the 10.13039/100000054NCI, 10.13039/100000002NIH, under contract no. HHSN26120150003I. We thank Swati Priya in the NCI for editorial assistance. We also thank Dr. M. Hong and Sal Fuerstenberg (Eureka) for critical review of the manuscript.

## Author contributions

M. Ho, as the guarantor of this study, conceived the research project and supervised the research. M. Ho and C.L. designed the studies. M. Huo, A.Q., and M. Ho wrote the manuscript. A.Q., M. Huo, D.L., L.E.H., M.S., C.R., J.O., D.A., R.Z., J.Z., and N.L. performed most of the experiments. J.C., J.L., and G.X. designed the AbTCR constructs and produced respective lentiviral vectors. A.Q., M. Huo, D.L., L.E.H., H.-E.T., E.E., H.Z., C.L., R.N., N.L., and M. Ho analyzed the data. All the authors reviewed, edited, and approved the manuscript.

## Declaration of interests

M. Ho and N.L. are inventors on the related patent no. WO2020033430 assigned to the NIH, “High affinity monoclonal antibodies targeting glypican-2 and uses thereof.” M. Ho, N.L., D.L., C.L., and H.Z. are the inventors on the related patent no. WO2023215738 assigned to the NIH and Eureka Therapeutics, “Compositions targeting GPC2 and GPC3 and their use for treating solid tumors.” C.L., H.Z., J.C., J.L., and G.X. are employees of Eureka Therapeutics.

## STAR★Methods

### Key resources table

**Table:** 

REAGENT or RESOURCE	SOURCE	IDENTIFIER
**Antibodies**		

Cetuximab	Lilly	NDC Code: 66733-948-23
Goat-*anti*-human IgG conjugated with PE	Jackson ImmunoResearch	Cat#109-116-170, RRID:AB_2337681
PE-conjugated anti-MYC mouse monoclonal antibody	Cell Signaling Technology (CST)	Cat#3739
Anti-human CD3-BV711	BioLegend	Cat#317328, RRID:AB_2562907
Anti-human CD3-BV605	BioLegend	Cat#317322, RRID:AB_2561911
Anti-human CD62L-APC	BioLegend	Cat#304810, RRID:AB_314470
Anti-human CD45RA-BV421	BD	Cat#569620
Anti-human LAG3-APC	BioLegend	Cat#369212, RRID:AB_2728373
Anti-human TIM3-PE-Cy7	BioLegend	Cat#345014, RRID:AB_2561720
Anti-human PD1-BV421	BioLegend	Cat#329920, RRID:AB_10960742
GPC2 (CT3) antibody	CST	Cat#90488
Anti-Phospho-PLCγ1	CST	Cat#2821S
Anti-PLCγ1	CST	Cat#2822S
Anti-Phospho-SLP76	CST	Cat#14745S
Anti-SLP76	CST	Cat#4958S
Anti-Phospho-LAT	CST	Cat#3584S
Anti-LAT	CST	Cat#45533S
Anti-Phospho-ZAP70	CST	Cat#2701S
Anti-ZAP70	CST	Cat#2705S
Anti-Phospho-mTOR	CST	Cat#5536
Anti-mTOR	CST	Cat#2983
Anti-GAPDH	CST	Cat#2118S
Anti-Histone H4	CST	Cat#2592S
Anti-NFκB p65	BioLegend	Cat#653002, RRID:AB_2561613
Anti-NFATc1	BioLegend	Cat#649602, RRID:AB_10679126
Anti-mouse IgG HRP-linked antibody	CST	Cat#7076P2
Anti-rabbit IgG HRP-linked antibody	CST	Cat#7074S
Anti-human CD3	Biocare	Cat#CME324B
Anti-human CD45RA	Invitrogen Life Technologies	Cat#14-0458-82, RRID:AB_467272
Anti-human CD8	CST	Cat#85336
Anti-human CD4	ABclonal	Cat#19018, RRID:AB_2862510
Anti-human CD27	Abcam	Cat#Ab131254, RRID:AB_11155136
Anti-human CD45RA	Abcam	Cat#Ab755, RRID:AB_305970

**Chemicals, peptides, and recombinant proteins**		

Human T-Activator CD3/CD28	Invitrogen	Cat#11131D

**Critical commercial assays**		

123count eBeads	Invitrogen	Cat#01-1234-42
ELISA MAX™ Deluxe Set Human IFN-γ	BioLegend	Cat#430104
Luciferase Assay System	Promega	Cat#E1501
PE Phycoerythrin Fluorescence Quantitation Kit	BD	Cat#340495
NE-PER Nuclear and Cytoplasmic Extraction Kit	ThermoScientific	Cat#78833
BCA protein assay	ThermoScientific	Cat#23225
LEGENDplex Human CD8/NK Panel V02	BioLegend	Cat#741187
CFP 4-plex kit	Spatomics	CFP Fluor 488/555/647/750

**Experimental models: Cell lines**		

Peripheral blood mononuclear cells	NIH Department of Transfusion Medicine	N/A
HEK-293T	ATCC	#CRL-3216
IMR5	NCI Pediatric Oncology Branch	N/A
SH-SY5Y	NCI Pediatric Oncology Branch	N/A
NBEB	NCI Pediatric Oncology Branch	N/A
LAN-1	NCI Pediatric Oncology Branch	N/A
Jurkat	ATCC	#TIB-152

**Experimental models: Organisms/strains**		

Mouse: NOD/SCID/IL-2Rgcnull (NSG) mice	NCI CCR Animal Resource Program/NCI Biological Testing Branch	RRID:BCBC_4142

**Software and algorithms**		

FlowJo	BD Biosciences	https://www.flowjo.com/flowjo/download
GraphPad Prism	Dotmatics	https://www.graphpad.com/features
ImageJ	NIH	https://imagej.net/ij/download.html
AlphaFold3	Abramson, Josh et al.^16^	https://alphafoldserver.com
Dali Server	Holm, Liisa et al.^23^	http://ekhidna2.biocenter.helsinki.fi/dali/
UCSF ChimeraX	Meng, Elaine C et al.^24^	https://www.cgl.ucsf.edu/chimerax/download.html
HALO	Indica labs	https://indicalab.com/halo/
Living Image Software	Revvity	https://www.revvity.com/product/li-software-for-lumina-1-seat-add-on-128110
Zeiss Zen software	ZEISS	https://www.zeiss.com/microscopy/us/products/software/zeiss-zen.html
FortéBio analysis software	Sartorius	https://www.sartorius.com

### Experimental model and study participant details

#### Human T cells

Human peripheral blood samples from healthy donors were provided by the NIH Clinical Center Department of Transfusion Medicine under the NIH Institutional Review Board–approved healthy donor program. Informed written consent was obtained from all healthy donors for the use of their blood samples for laboratory research purposes by NIH researchers. The donors’ gender and age are unknown. Donor peripheral blood mononuclear cells (PBMCs) were isolated using Ficoll Tubes (Greiner Bio-One). Cells were stimulated with Human T-Activator CD3/CD28 (Invitrogen) at a 2:1 bead-to-T cell ratio in RPMI with 50 U/mL of IL-2. The activated T cells were transduced with the previously concentrated lentivirus. The transduction efficiency and binding to GPC2 were measured on day 8. Cells were subsequently used for *in vitro* cell killing assays, *in vivo* mice studies, or frozen down on day 10.

#### Cell lines

HEK-293T (originally from ATCC #CRL-3216) cell lines were obtained from Dr. Ira Pastan at the NCI. The HEK-293T cells were cultured in DMEM with 10% FBS, 1% L-glutamine, and 1% penicillin–streptomycin. Jurkat cells were purchased from ATCC. The tdTomato (reporter gene) was engineered by our lab to be downstream of the NFAT and NF-κB site in the Jurkat T cell line. Neuroblastoma cell lines, including IMR5, SH-SY5Y, NBEB, and LAN-1, were obtained from the NCI Pediatric Oncology Branch (Bethesda, MD). IMR5 and LAN-1 cell lines expressing firefly luciferase-p2A-GFP (GL) were generated in our lab. The NBEB cell line, which expresses luciferase-p2A-mCherry (ML), was obtained from Brad St Croix at the NCI. IMR5 and SH-SY5Y cell lines expressing firefly luciferase-p2A-GFP (GL), utilized in the orthotopic mouse models, were provided by Dr. Rosa Nguyen at the NCI. RPMI 1640 medium with 10% FBS, 1% L-glutamine, and 1% penicillin-streptomycin was used to culture neuroblastoma cell lines. All cells were maintained at 37°C in a humidified atmosphere with 5% CO2. All cell lines were authenticated by morphology and growth rate and were mycoplasma-free.

#### Animal studies

All mice were housed and treated under the protocol (LMB-059) approved by the Institutional Animal Care and Use Committee at the NIH. NCI-Frederick is accredited by AAALAC International and follows the Public Health Service Policy for the Care and Use of Laboratory Animals. Animal care was provided in accordance with the procedures outlined in the ‘Guide for Care and Use of Laboratory Animals (National Research Council; 1996; National Academy Press; Washington, D.C.)’.

Female NOD/SCID/IL-2Rgc^null^ (NSG) mice (NCI CCR Animal Resource Program/NCI Biological Testing Branch), which are immunodeficient and lack mature T cells, B cells, and natural killer cells, were housed at five-week old and treated under the protocol (LMB-059) approved by the Institutional Animal Care and Use Committee at the NIH. Mice were housed under pathogen-free conditions. In the IMR5-GL intravenous (i.v.) study, mice were i.v. injected with 4 million IMR5-GL cells. Tumors were allowed to be established for 4–5 weeks, and mice were randomly assigned to groups. In the initial study comparing the CT3 and hCT3 CAR T cell variants, mice were randomly assigned to six groups consisting of CT3, hCT3-1, hCT3-2, hCT3-3, hCT3-4, and mock groups, which received 5 million IMR5-GL cells. Then, the mice were i.v. infused with 10 million CAR T or mock T cells. The number of infused CAR or AbTCR T cells was calculated based on transduction efficiency to ensure a consistent dose of transgene-positive cells. In the mock group, an equivalent number of total mock T cells was infused to match the total number of T cells used in the treatment groups. In the following IMR5-GL study to compare functions of CAR and AbTCR T cells, mice were treated with 5 million effective CAR and AbTCR T cells when the tumor reached 1 × 10^9^ photons/sec/cm^2^/sr. Tumor re-challenge was conducted in three hCT3 AbTCR T cell-treated mice and five untreated control mice.

The NBEB and LAN1 mouse models were established by subcutaneously injecting 2.5 million NBEB-ML and 3 million LAN1-GL cells, respectively. The orthotopic mouse models were created by performing a para-adrenal injection of 0.5 million IMR5-GL or SH-SY5Y-GL cells.

### Method details

#### Generation of AbTCR T cell and CSR constructs

For the AbTCR T cell receptor, VL of anti-GPC2 antibody (CT3 or hCT3) is fused to the human TCRγ constant region and VH of CT3 or hCT3 is fused to that of TCRδ. The chimeric stimulating receptor (CSR) consists of an anti-GPC2 scFv (CT3 or hCT3) fused to the hinge, transmembrane, and intracellular domains of CD30 molecule. These constructs are cloned into lentiviral expression vectors and used to produce the lentiviruses.

#### Production of lentivirus

Lentivirus was produced by transfecting HEK-293T cells with GPC2-CAR or GPC2-AbTCR+CSR lentiviral vectors, along with a packaging plasmid (PAX2, Plasmid #12260, Addgene) and an enveloping plasmid (MD2G, Plasmid #12259, Addgene). Transfection was performed using the CalFectin transfection reagent (SignaGen). Lentivirus was harvested 72 h after transfection and subsequently concentrated using the Lenti-X concentrator (TakaraBio). HEK-293T or Jurkat cells were transduced using the concentrated lentivirus to measure the lentivirus titer. The HEK-293T or Jurkat cells were stained with antibodies and fluorochromes corresponding to the tEGFR tag on the CAR T cells or the MYC tag on the AbTCR T cells. The HEK-293T cells or Jurkat were then analyzed by flow cytometry, and a titer was calculated.

#### Mouse tumor imaging

In all mouse studies, the mice were monitored regularly and imaged using 3 mg D-luciferin (PerkinElmer). The mice were intraperitoneally (i.p.) injected with D-luciferin and imaged 10 min later using a Xenogen IVIS Lumina (PerkinElmer). The Living Image Software was used to collect images and analyze the bioluminescence signal flux for the mice as photons per second per square centimeter per steradian (photons/s/cm^2^/sr). The endpoint was set as tumor volume exceeding 1,500 mm^3^ or onset of distress symptoms, which include typical signs such as hunching, lethargy or reduced activity, labored breathing, ruffled fur or dehydration, poor grooming, reduced food or water intake, inability to ambulate or reach food, and scratching or bleeding tumors.

#### Flow cytometry

Multiple experiments were conducted through flow cytometry using the SONY ID7000 (Sony Biotechnology) and the FlowJo software (BD Biosciences). The lentiviral transduction efficiencies of the CAR T cells were measured by staining the cells with anti-EGFR human monoclonal antibody cetuximab (Erbitux) and a goat-*anti*-human IgG conjugated with PE (Jackson ImmunoResearch). Additionally, the transduction efficiency of the AbTCR T cells was measured by staining the cells with a PE-conjugated anti-MYC mouse monoclonal antibody (Cell Signaling Technology). The GPC2 binding of both the CAR T cells and AbTCR T cells was assessed by staining the cells with human GPC2 tagged with a human Fc (Acrobiosystems) at a concentration of 3 μg/mL and subsequently staining the cells with a goat-*anti*-human IgG conjugated with PE. The secondary antibody-only control (cells stained with the secondary antibody in the absence of the primary antibody) served as the Mock group to account for any nonspecific background signal.

The T cell memory phenotype within mouse blood was analyzed using anti-human CD3-BV711 (BioLegend), anti-human CD62L-APC (BioLegend) and anti-human CD45RA-BV421 (BD). Analyses of T cell exhaustion within mice blood were performed using anti-human CD3-BV605, anti-human LAG3-APC, anti-human TIM3-PE-Cy7, anti-human PD1-BV421 from BioLegend.The FACS plots generated for mock, CAR, and AbTCR T cells used CD3^+^, CD3+EGFR+, and CD3+MYC+, respectively. Additionally, 123count eBeads (ThermoFisher) were used according to the manufacturer’s protocol to quantify the number of CAR T and AbTCR T cells per microliter of blood.

#### Structure models of antibodies

The structure models of the CT3 antibody and its four humanized forms, hCT3-1, hCT3-2, hCT3-3, and hCT3-4, were generated by the AlphaFold3 server^16^ and aligned with Dali Server.^23^ The structure models were analyzed and rendered with UCSF ChimeraX.^24^ The VH and VL regions of the humanized CT3 models are highlighted in pink and yellow, respectively. The combined CDR1, CDR2, and CDR3 regions (KABAT/IMGT/Paratome) are labeled in green, cyan, and magenta, respectively. Z-scores represent the sum of equivalent residue-wise Cα-Cα distances, and overall RMSD values of the aligned structures for each VH and VL are listed. RMSD for local residues is illustrated on a blue-red color scale from 0.01–1.20 Å.

#### *In vitro* cytotoxicity assays and ELISA

To assess cytotoxicity, CAR, AbTCR, or mock T cells were co-cultured with 2,000 luciferase-expressing neuroblastoma target cells (including IMR5, NBEB, and LAN-1) at defined effector-to-target (E:T) ratios (typically 0.625:1, 1.25:1, 2.5:1, 5:1, 10:1, or 20:1) in triplicate wells without the addition of exogenous interleukins. The number of effector cells added to each well was adjusted based on transduction efficiency, as determined by flow cytometry (e.g., MYC-tag or hEGFRt expression). For example, if transduction efficiency was 50%, twice the number of total T cells were added to achieve the intended number of CAR^+^ or AbTCR^+^ T cells per well. Co-cultures were maintained for 24, 48, or 72 h, depending on the experimental design. At the indicated time points, supernatants were collected and stored at −80°C for subsequent cytokine analysis. IFN-γ levels in the supernatants were quantified using the ELISA MAX Deluxe Set Human IFN-γ (BioLegend), following the manufacturer’s instructions. After cytokine collection, the remaining adherent or suspension tumor cells were lysed in Passive Lysis Buffer (Promega) for 15 min at room temperature.

Tumor cell viability was assessed by measuring luciferase activity using the Luciferase Assay System (Promega) and a SpectraMax plate reader. Luminescence readings were normalized to target-only control wells (no T cells), and percent specific lysis was calculated using the following formula:

%Specificlysis=[1−(RLU_sample/RLU_target−only)]×100

where RLU represents relative luminescence units. Data were analyzed from three independent experiments, and results are presented as mean ± standard deviation (SD).

#### T cell activation and exhaustion assays

NBEB tumor cells were co-cultured with T cells in a 1:1 ratio for 0, 2, 6, and 24 h. We collected the supernatant and centrifuged it at 250 rcf for 5 min to isolate T cells for Western blot.

To assess the proportion of exhausted T cells after prolonged antigen exposure, we co-cultured NBEB and T cells in a 1:1 ratio on Day 0. We transferred T cells to newly passaged NBEB cells with a 1:1 ratio every 48 h. On Day 8 and Day 14, we collected the T cells for CAR/AbTCR^+^PD1^+^ detection using flow cytometry.

#### NFAT/NFκB reporter assay

Engineered NFAT/NFκB-tdTomato Jurkat cells were transduced with CAR and AbTCR+CSR. At day 7, the transduction efficiencies were measured and then co-cultured with GFP-overexpressing IMR5 cells at the E:T ratio of 1:1 for 6 and 24 h using a poly-L-lysine-coated 8-well glass-bottom slide (ibidi). Images were then taken on a Zeiss LSM 880 Airyscan confocal microscope (Zeiss). tdTomato expression level was measured through flow cytometry and quantified by FlowJo.

#### GPC2 quantification in neuroblastoma cells

Neuroblastoma cell lines were stained with the (GPC2) CT3 mouse antibody (Cell Signaling Technology) and a secondary goat-*anti*-human IgG conjugated with PE antibody (Jackson ImmunoResearch) according to the recommended dilution. The antibodies bound per cell were estimated through flow cytometry using a PE quantification kit (BD Quantibrite PE Phycoerythrin Fluorescence Quantitation Kit).

#### Western blotting

Nuclear and cytosolic proteins were extracted from CAR and AbTCR T cells using the NE-PER Nuclear and Cytoplasmic Extraction Kit following the manufacturer’s protocol. Protein concentrations were measured using the BCA protein assay (ThermoScientific). 10 μg protein samples with Laemmli Sample Buffer (Bio-Rad) were subjected to electrophoresis on 4–20% SDS/PAGE gels (Invitrogen) and transferred to PVDF transfer membranes. After being blocked in 5% nonfat dry milk (Bio-Rad) at room temperature, the membranes were incubated with primary antibodies overnight at 4°C. The primary antibodies included anti-Phospho-PLCγ1 (#2821S), anti-Phospho-SLP76 (#14745S), anti-Phospho-LAT (#3584S), anti-Phospho-ZAP70 (#2701S), anti-Phospho-mTOR (#5536), anti-GAPDH (#2118S), anti-Histone H4 (#2592S), anti-PLCγ1 (#2822S), anti-SLP76 (#4958S), anti-ZAP70 (#2705S), anti-mTOR (#2983), and anti-LAT (#45533S) from Cell Signaling Technology; and anti-NFATc1 (#649602) and anti-NFκB p65 (#653002) from Biolegend. The anti-mouse IgG HRP-linked antibody (Cell Signaling Technology #7076P2) and anti-rabbit IgG HRP-linked antibody (Cell Signaling Technology #7074S) were used as the secondary antibodies.

For quantification of phosphorylation levels, densitometric analysis was conducted using ImageJ software. The intensity of each phospho-protein band was first normalized to its corresponding total protein band from the same lane (e.g., phospho-ZAP70 to total ZAP70, phospho-LAT to total LAT, etc.) to control for loading differences. Subsequently, normalized values for each experimental group were further normalized to the mean value of the mock group, which was set as 1. This allowed calculation of the relative fold-change in phosphorylation compared to baseline. All densitometric analyses were performed on non-saturated exposures to ensure quantitative accuracy, and statistical comparisons were based on biological replicates.

#### Plasma cytokine panel

Mice blood was collected at weeks 2–4 after infusion of the CAR or AbTCR T cells using the submandibular blood collection technique. 100 μL of blood was collected in EDTA-coated tubes (Greiner Bio-One) to prevent clotting, followed by lysing using red blood cell lysis buffer (eBioscience). Mice plasma was subsequently isolated and used for cytokine analysis using the LEGENDplex Human CD8/NK Panel V02 (BioLegend) according to the manufacturer’s protocol. The isolated blood cells were further analyzed through flow cytometry.

#### Immunohistochemistry and pathology analysis

Tumors were fixed in 10% neutral buffered formalin, routinely processed, embedded, and sectioned into 5 μm slices. These slides were baked at 60°C for one hour before staining with chromogenic immunohistochemistry (IHC) using the Leica Biosystems Bond-Max automated stainer. The heat-induced epitope retrieval in EDTA was used to stain for human CD3 (Biocare #CME324B, rabbit monoclonal antibody, 1:50), human GPC2 (CT3, Cell Signaling Technology #90488, mouse monoclonal antibody, 1:100), and human CD45RA (Invitrogen Life Technologies, #14-0458-82, 1:2000). The positively stained cells represent the transferred T cells. Positive tissue controls included human neuroblastoma for CT3/GPC2 and human tonsil for CD3 and CD45RA. IHC isotype-negative controls were performed by replacing the primary antibody with a non-clonal, isotype-matched antibody from the same species.

Slides were digitized at 20× objective (0.5× 0.5 μm per pixel) using the Aperio AT2 scanner (Leica Biosystems) and analyzed with HALO software (Indica Labs, v3.6). A board-certified pathologist confirmed the appropriateness of staining and annotated regions of interest to include viable tumor tissue while excluding necrosis, non-tumor tissues, and artifacts. Tumor positivity and immune cell infiltrates were quantified using image analysis, which included identifying stain vectors, optimizing cell detection algorithms, and thresholding chromogenic IHC staining based on positive and negative controls. CT3/GPC2 staining exhibited a membranous to cytoplasmic pattern; for quantification, cells were categorized based on staining intensity as negative, 1+, 2+, or 3+. The percentage of positive cells was determined for each category, and an H-score was calculated. CD3 staining was quantified by counting the number of positive cells per mm^2^. CD45RA staining was quantified similarly.

#### Immunofluorescence staining of GPC2 and CD3

Tumors were fixed in 10% neutral buffered formalin, routinely processed, embedded, and sectioned into 5 μm slices. These sections were stained using the Leica Biosystems Bond-Max automated stainer with multiplex immunofluorescence for human CD3 (Biocare #CME324B, EDTA HIER, 1:50) and human GPC2 (CT3, CST #90488, EDTA HIER, 1:100), utilizing Opal 520 and Opal 570 fluorophores, respectively. DAPI was used for nuclear staining, and Gold antifade mounting media was used with coverslips. Whole slide fluorescent imaging was performed with an Akoya PhenoImager HT scanner using a 20× objective (0.5× 0.5 μm per pixel). Positive tissue controls included human tonsil and neuroblastoma tissues. Negative controls involved replacing primary antibodies with isotype-matched antibodies from the same species.

#### Multiplexed immunofluorescence

Formalin-fixed paraffin-embedded (FFPE) slides were baked in an oven at 60°C for 2 h. Subsequent assay steps were performed using the Biocare Oncore Pro X automated staining system. After dewaxing, antigen retrieval, washing, and blocking, the tissue sections were incubated with primary antibodies targeting human CD8 (CST, 85336), CD4 (ABclonal, 19018), CD27 (Abcam, Ab131254), and CD45RA (Abcam, Ab755), followed by incubation with anti-mouse or anti-rabbit HRP-conjugated secondary antibodies (Invitrogen, B40961/B40962) in combination with the CFP 4-plex kit (Spatomics, CFP Fluor 490/555/645/750). Tissue imaging was conducted at 20× using a Zeiss Axio Observer microscope and analyzed with Zeiss Zen software.

#### Octet analysis

The binding kinetics of the CT3 and humanized CT3 antibodies to GPC2 were determined using the Octet RED96 system (FortéBio). Antibodies were loaded at concentrations of 200 nM, 100 nM, and 50 nM. All experiments were performed at 30°C, and reagents were prepared in PBS (pH 7.4) with 0.1% BSA and 0.1% Tween 20. Biotinylated GPC2–hFc protein (1 μg/mL) was immobilized onto streptavidin biosensors, which were subsequently used in association and dissociation measurements for a time window of 180 s and 300 s, respectively. Data analysis was performed using the FortéBio analysis software provided with the instrument.

### Quantification and statistical analysis

Flow cytometry was performed using Sony ID7000 and analyzed using FlowJo. Densitometric analysis for Western blotting was performed using ImageJ software. Xenogen IVIS Lumina (PerkinElmer) was used for mouse model studies and the Living Image Software was used to analyze the bioluminescence signal flux. Confocal images were taken on a Zeiss LSM 880 Airyscan confocal microscope (Zeiss). Tumor regression was assessed using a ‘change to the baseline’ analysis. Data were analyzed using one-way ANOVA with Tukey’s multiple comparisons test, two-tailed unpaired Student’s t test, or two-way ANOVA with Tukey’s multiple comparisons test with GraphPad Prism and presented as mean ± SEM. *p* < 0.05 or *p* < 0.01 were considered statistically significant.

## Supplemental information

Document S1. Figures S1–S5

Document S2. Article plus supplemental information
