# Supplementary material for: Antibody-gamma/delta T cell receptors targeting GPC2 regress neuroblastoma with low antigen density
Source: Cell Rep Med. 2025 Sep 29;6(10):102378. doi: 10.1016/j.xcrm.2025.102378 (PMC12629815; doi:10.1016/j.xcrm.2025.102378)
Supplement: Document S1. Figures S1–S5 [file mmc1.pdf]

Cell Reports Medicine, Volume *6*

Supplemental information

Antibody-gamma/delta T cell receptors targeting GPC2 regress neuroblastoma with low antigen density

Alex Quan, Mingyu Huo, Dan Li, Laura E. Hutchins, Constanza Rodriguez, Jangsuk Oh, Hsi-En Tsao, Madeline Spetz, Elijah Edmondson, Dana Ashworth, Rui Zheng, Jing Zhou, Jinyun Chen, Jingbao Liu, Guangyan Xiong, Hongbing Zhang, Cheng Liu, Rosa Nguyen, Nan Li, and Mitchell Ho

```
CT3      1   EVQLQQSGPELVKPGASVKMSCKASRFTFTDYNIHWVKQSPGKTLEWIGYINPNNGDIFYKQKFNGKATL
hCT3-1   1   QVQLVQSGAEVKKPGASVKVSCKASRFTFTDYNIHWVRQAPGQGLEWIGYINPNNGDIFYKQKFNGRVTL
hCT3-2   1   QVQLVQSGAEVKKPGASVKVSCKASRFTFTDYNIHWVRQAPGQRLEWIGYINPNNGDIFYKQKFNGRVTI
hCT3-3   1   QVQLVQSGAEVKKPGASVKVSCKASRFTFTDYNIHWVRQAPGQGLEWIGYINPNNGDIFYKQKFNGKATM
hCT3-4   1   QVQLVQSGAEVKKPGASVKVSCKASRFTFTDYNIHWVRQAPGQRLEWIGYINPNNGDIFYKQKFNGRVTI

CT3      71  TINKSSNTAYMELRSLTSEDSAVYYCVRSSNIRYTFDRFFDVWGTGTTVTVSSGGGGSGGGGSGGGGS-E
hCT3-1   71  TADKSTSTAYMELSSLTSEDTAVYYCVRSSNIRYTFDRFFDVWGQGTLVTV-SGGGGSGGGGSGGGGS-D
hCT3-2   71  TRDTSASTAYMELSSLRSEDTAVYYCVRSSNIRYTFDRFFDVWGQGTLVTV-SGGGGSGGGGSGGGGS-D
hCT3-3   71  TVDTSTSTVYMELSSLRSEDTAVYYCVRSSNIRYTFDRFFDVWGQGTLVTV-SGGGGSGGGGSGGGGSMD
hCT3-4   71  TRDTSASTAYMELSSLRSEDTAVYYCVRSSNIRYTFDRFFDVWGQGTLVTV-SGGGGSGGGGSGGGGS-E

CT3      140 NVLTQSPAIMSASLGEKVTMSCRASSSVNYIYWYQQKSDASPKLWIYYTSNLAPGVPARFSGSGSGNSYS
hCT3-1   139 VVMTQSPLSLPVTPGEPASISCRASSSVNYIYWYLQKPGQSPQLWIYYTSNLAPGVPDRFSGSGSGTDFT
hCT3-2   139 VVMTQSPAFLSVTPGEKVTITCRASSSVNYIYWYQQKPDQAPKLWIYYTSNLAPGVPSRFSGSGSGTDFT
hCT3-3   140 IQMTQSPSSLSASVGDRVTITCRASSSVNYIYWYQQKSGKAPKLWIYYTSNLAPGVPSRFSGSGSGTDFT
hCT3-4   139 IVLTQSPATLSLSPGERATLSCRASSSVNYIYWYQQKPGQAPRLWIYYTSNLAPGIPARFSGSGSGTDFT

CT3      210 LTISSMEGEDAATYYCQQFSSSPSTFGTGTKLELK
hCT3-1   209 LKISRVEAEDVGVYYCQQFSSSPSTFGQGTKLEIK
hCT3-2   209 FTISSLEAEDAATYYCQQFSSSPSTFGQGTKLEIK
hCT3-3   210 LTISSLQPEDFATYYCQQFSSSPSTFGQGTKLEIK
hCT3-4   209 LTISSLEPEDFAVYYCQQFSSSPSTFGQGTKLEIK
```

(yellow)	Differences in amino acids between hCT3 and CT3
(red)	Complementarity Determining Regions (CDRs)
(blue)	Linker

Figure S1. Sequence alignment of humanized CT3 scFv, related to Figure 1
Differences in amino acids between CT3 and HCT3 are highlighted in yellow. The complementarity determining regions are marked in red, and the linkers are highlighted in blue.

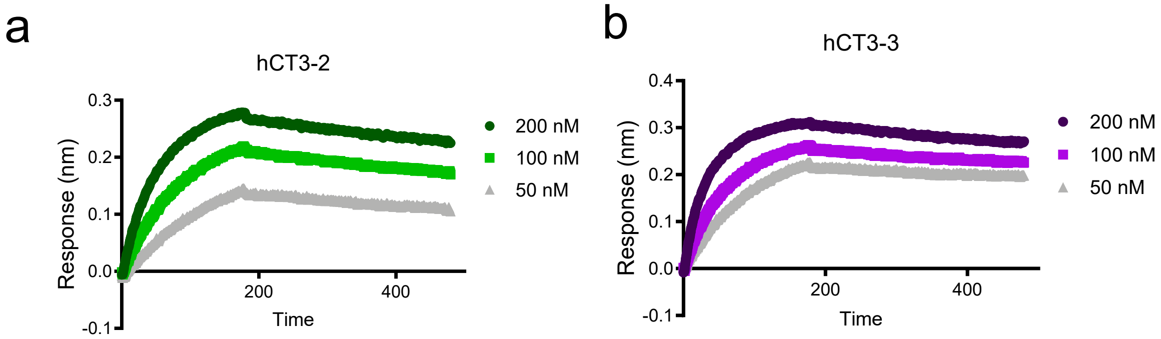

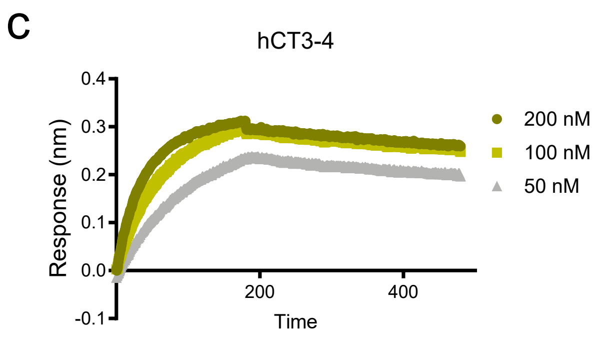

d

P-values of in vitro assay	3:1	6.25:1	12.5:1
CT3 vs Mock	0.0165	0.0013	0.0001
hCT3-1 vs CT3	0.0016	0.0110	0.0011
hCT3-2 vs CT3	0.0080	0.0186	0.0038
hCT3-3 vs CT3	0.0092	0.0034	0.0021
hCT3-4 vs CT3	0.0001	0.0018	0.0009

Figure S2. Validation of hCT3 mAb function, related to Figure 1
Octet analysis of GPC2 binding of hCT3-2 (a), hCT3-3 (b), and hCT3-4 (c) antibodies at concentrations of 50 nM, 100 nM, and 200 nM. (d) Statistical results of the killing assay shown in Fig. 1f.

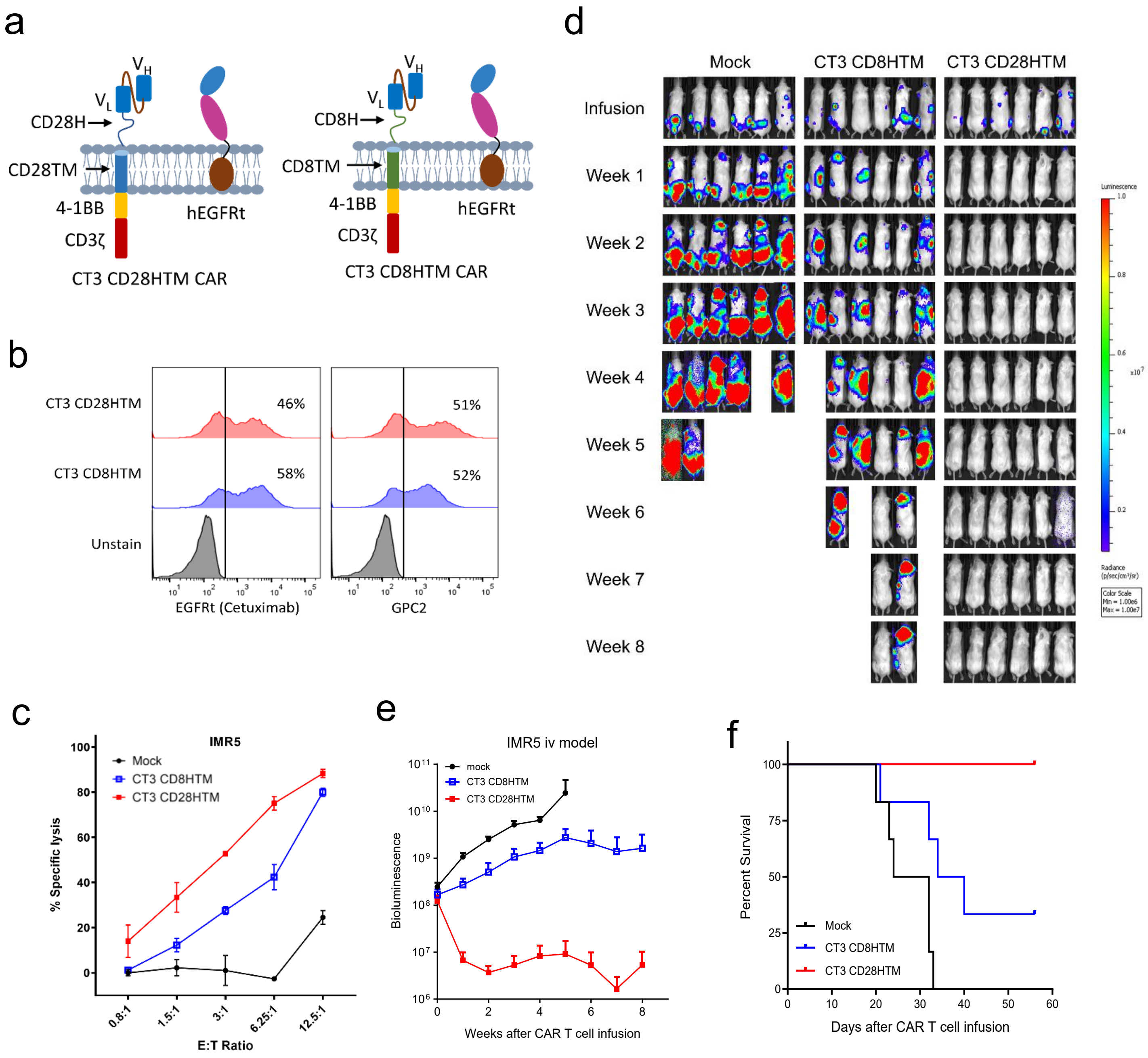

Figure S3. Comparison of CAR constructs with CD8HTM and CD28HTM, related to Figure 2
(a) Schematics of the GPC2-targeted CAR constructs with CD8HTM and CD28HTM. (b) CAR expression and GPC2 binding of CT3 CD8HTM CAR and CT3 CD28HTM CAR T cells. (c) Cytolytic activity of CT3 CD8HTM CAR and CT3 CD28HTM CAR T cells against IMR5 at various E: T ratios. n = 3 independent experiments. (d-e) Mice were treated with 10 million CAR T cells when tumor burden reached approximately 1×10^8 photons/sec. Representative bioluminescence images of IMR5 tumor-bearing mice (d) and tumor growth curves (e) measured using IVIS imaging. (f) Survival rate of the mouse model. n = 6 mice per group.

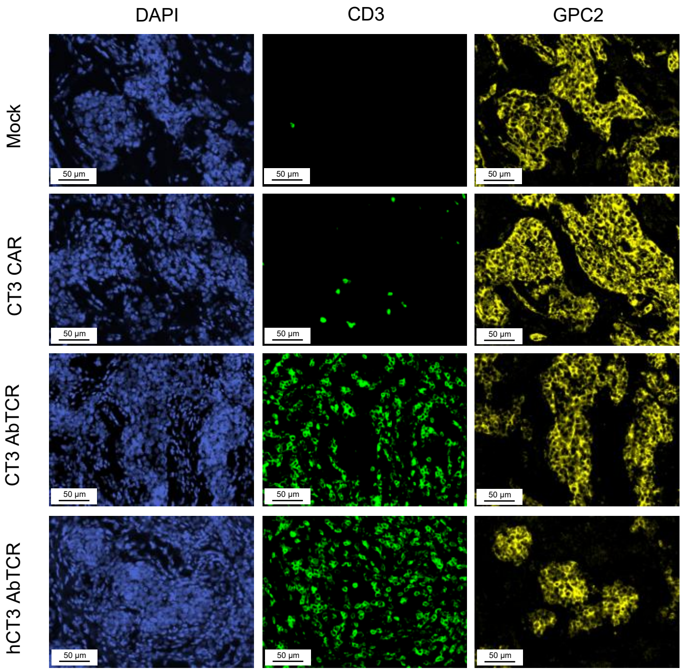

Figure S4. Single channel images of immunofluorescence, related to Figure 4
Scale bar: 50 µm.

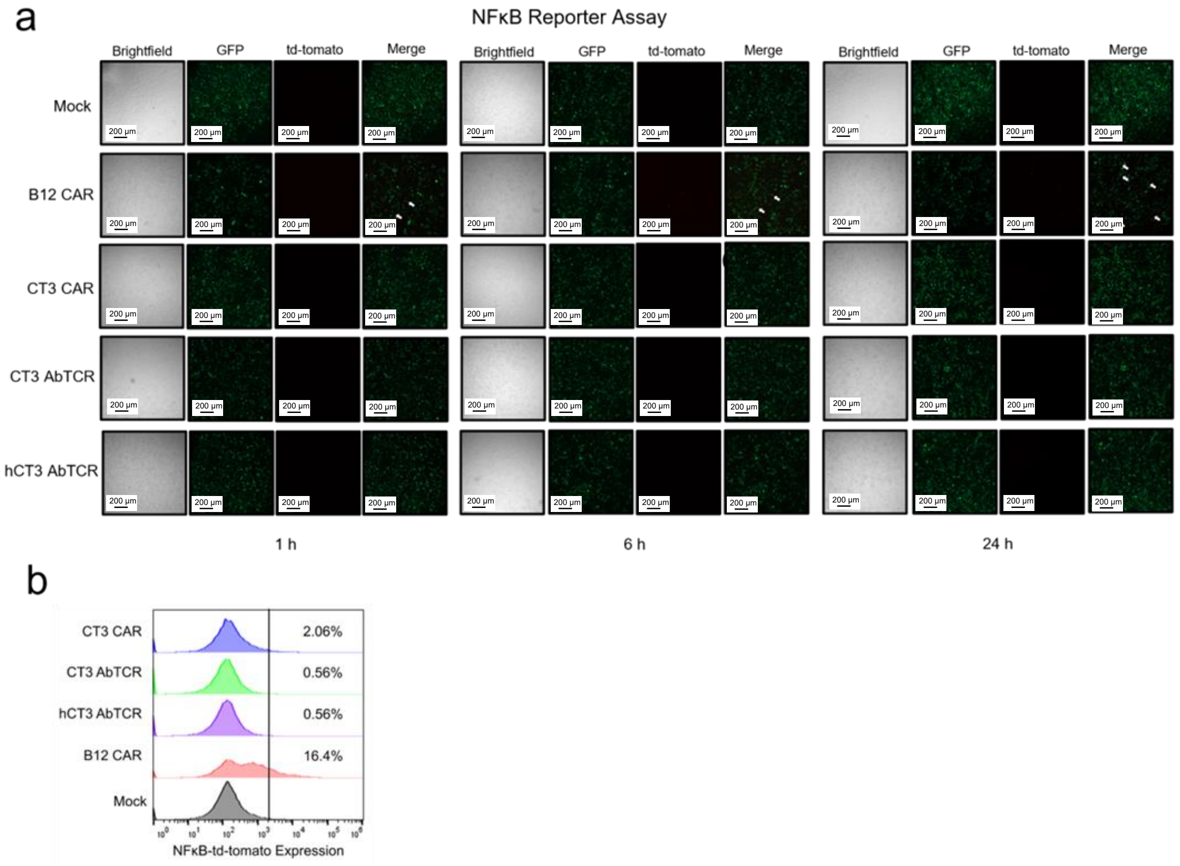

Figure S5. NFκB reporter assay in Jurkat cells, related to Figure 6
(a) Confocal microscopy of NFκB Jurkat reporter cells interacting with IMR5 tumor cells after 1, 6, and 24 hours of incubation. Scale bar is 200 µm. (b) NFκB-td-tomato expression levels measured by flow cytometry.
